# *GhFAD2–3* is required for anther development in *Gossypium hirsutum*

**DOI:** 10.1186/s12870-019-2010-9

**Published:** 2019-09-10

**Authors:** Feng Liu, Lihong Ma, Youwu Wang, Yanjun Li, Xinyu Zhang, Fei Xue, Xinhui Nie, Qianhao Zhu, Jie Sun

**Affiliations:** 1 Key Laboratory of Oasis Eco-agriculture, College of Agriculture, Shihezi University, Shihezi, Xinjiang, 832000 China; 2 College of Plant Science and Technology, Tarim University, Alar, Xinjiang, 843300 China; 3 CSIRO Agriculture and Food, GPO Box 1700, Canberra, 2601 Australia

**Keywords:** *Gossypium hirsutum*, Fatty acid composition, *GhFAD2*, Male sterile, Linoleic acid, Oleic acid

## Abstract

**Background:**

In higher plants, the *FAD2* gene encodes the microsomal oleate ^Δ12^-desaturase, one of the key enzymes essential for the biosynthesis of the polyunsaturated lipids that serve many important functions in plant development and stress responses. FAD2 catalyzes the first step, in the biosynthesis of the polyunsaturated fatty acids (PUFAs) found in the cell membrane and cell wall, and it is thus of great importance to investigate the regulatory role of *FAD2* in anther development.

**Results:**

We reported the molecular characterization of the cotton (*Gossypium hirsutum*) *GhFAD2* gene family and the essential role of *GhFAD2–3* in cotton anther development. *G. hirsutum* contains four pairs of homoeologous *FAD2* genes (*GhFAD2–1* to *GhFAD2–4*). *GhFAD2–3* is ubiquitously and relatively highly expressed in all analyzed tissues, particularly in anthers. Specific inhibition of *GhFAD2–3* using the RNA interference approach resulted in male sterility due to impaired anther development at the stages from meiosis to maturation. The cellular phenotypic abnormality observed at the meiosis stage of the *GhFAD2–3* silenced plant (*fad2–3*) coincides with the significant reduction of C18:2 in anthers at the same stage. Compared with that of the wild type (WT), the content of C18:1 was 41.48%, which increased by 5 fold in the *fad2–3* anther at the pollen maturation stage. Moreover, the ratio of monounsaturated to polyunsaturated fatty acid was 5.43 in *fad2–3* anther, which was much higher than that of the WT (only 0.39). Through compositional analysis of anthers cuticle and transcriptome data, we demonstrated it was unfavorable to the development of anther by regulating *GhFAD2–3* expression level to increase the oleic acid content.

**Conclusions:**

Our work demonstrated the importance of C18:2 and/or C18:3 in the development of the pollen exine and anther cuticle in cotton and provided clue for further investigation of the physiological significance of the fatty acid composition for plant growth and development.

**Electronic supplementary material:**

The online version of this article (10.1186/s12870-019-2010-9) contains supplementary material, which is available to authorized users.

## Background

In higher plants, the microsomal oleate ^Δ12^-desaturase (fatty acid desaturase 2, FAD2) is a hydrophobic endoplasmic reticulum protein, catalyzing the reaction from monounsaturated oleic acid (C18:1) to polyunsaturated linoleic acid (C18:2) by introducing a double bond between the 12th and 13th carbon atoms of C18:1 [1]. *FAD2* was first identified in the model plant species *Arabidopsis thaliana* [2]. Although only a single copy of *FAD2* was found in *A. thaliana*, multiple copies of *FAD2* were identified in many other plants, such as canola (*Brassica napus*), cotton (*Gossypium hirsutum*), soybean (*Glycine max*), olive (*Olea europaea*), sesame (*Sesamum indicum*) and sunflower (*Helianthus annuus*) [3–8].

As the key gene controlling the conversion of C18:1 to C18:2, the *FAD2* transcriptional level directly determines the relative content and proportion of polyunsaturated fatty acids (PUFAs) in plants [9]. *FAD2* could be significantly induced by abiotic stresses [10, 11], suggesting that PUFAs could play a key role in plant stress responses and adaptation to environmental change. Due to the oxidative instability of PUFAs, studies on the regulation of *FAD2* were mainly focused on reducing its expression level to decrease the C18:2 content in oil seeds, and thus to improve oil quality [4, 12–16].

In flowering plants, the anther cuticle and pollen wall are rich in lipids, mainly fatty acids and their derivatives [17–21]. Genetic analyses of *Arabidopsis* and rice have found that many genes with an important role in the development of the anther cuticle and pollen wall are involved in lipid metabolism, such as *MALE STERILITY 2* (*MS2*), *FACELESS POLLEN 1* (*FLP1*), *CYP703*, *CYP704B1, CYP704B2*, *Acyl-CoA Synthetase 5* (*ACOS5*), *NO EXINE FORMATION1* (*NEF1*), *Wax-Deficient Anther1* (*WDA1*), *β-ketoacyl-coenzyme A synthase ECERIFERUM6* (*CER6*), *Defective Pollen Wall* (*DPW*), *Fatty acyl-coenzyme A Reductase* (*FAR*), *3-ketoacyl-CoA Synthase 9* (*KCS9*), and *OsC6* [18, 22–34].

The upland cotton genome is large and complex allotetraploid (AADD; 2n = 52) and several rounds of genome duplication events have been identified in cotton genome [35], which makes it more difficult to analyze gene expression and regulation. In this study, based on the characterization of the cotton (*G. hirsutum*) *FAD2* family genes, we investigated their expression patterns in various vegetative and reproductive tissues and found that *GhFAD2–3* is the gene highly expressed in most tissues analyzed, particularly in anthers. We demonstrated that silencing of *GhFAD2–3* resulted in male sterility, due to nonviable pollen grains and abnormal anther development resulted from significantly reduced levels of PUFAs at the meiosis and tetrad stages. This is also the first report on the dynamic changes of fatty acid constituents during cotton anther development, which were exactly opposite to those in developing and mature cottonseeds. Our work showed the effect of changes in fatty acid constituents on the physiological activity of anthers and revealed the essentialness of primary PUFAs in the development of the pollen wall and anther cuticle in cotton.

## Results

### Genome-wide identification of *GhFAD2* in cotton

The protein sequence of a previously identified *GhFAD2* gene (GenBank acc. no. X97016) was used to BLASTP the annotated proteins of *G. hirsutum* [35], *G. raimondii* [36] and *G. arboreum* [37]. Four, five and nine putative *FAD2* genes were identified in *G. arboreum* (Ga), *G. raimondii* (Gr) and *G. hirsutum* (Gh), respectively. Based on phylogenetic analysis, each of the four Ga putative *FAD2* and each of the five Gr putative *FAD2* has a corresponding copy in the At and Dt subgenomes of Gh, respectively (Additional file 1: Figure S1), suggesting that the *FAD2* gene family is highly conserved during the evolutionary history of cotton. Two Gr putative FAD2 on chromosome 13 (Gorai.013G248700 and Gorai.013G248800) are next to each other and Gorai.013G248700 lacks the 3rd conserved histidine-cluster observed in all plant FAD2 proteins [7]. We thus considered *Gorai.013G248700* as a non-authentic (or pseudogenized) *FAD2* gene. This observation suggests that *Gorai.013G248700* could be a result of gene duplication followed by pseudogenization. Interestingly, a similar situation was observed in the two Gh orthologs (*Gh_D13G2237* and *Gh_D13G2238*) of the two Gr genes. We therefore disregarded *Gh_D13G2237* as an *FAD2* gene and only used the remaining eight in the following analysis. The annotated *GhFAD2–4A* (*Gh_A01G2091*) is incomplete due to a sequence gap. We completed its cDNA sequence based on our RNA-seq data. Of these eight genes, five have previously been cloned. We renamed these eight *GhFAD2* genes with the aim of keeping the previous nomenclature of the five cloned genes as intact as possible (Table 1).

**Table 1:** The *GhFAD2* genes in *Gossypium hirsutum*

Gene name	Also known as	Locus ID	Chromosome and coordinates	GenBank accession no.	CDS length (bp)	No. of amino acid
GhFAD2–1A	FAD2–1	Gh_A13G1850	A13:78167608..78168765	X97016	1158	385
GhFAD2–1D	FAD2–1	Gh_D13G2238	D13: 58471954..58473105	HQ259410	1152	383
GhFAD2–2A	/	Gh_A01G2094	A01:23332066..23333199	/	1134	377
GhFAD2–2D	FAD2–2	Gh_D01G1227	D01:30322983..30328022	Y10112	1134	377
GhFAD2–3A	FAD2–3	Gh_A11G2814	A11:91511504..91512658	AF331163	1155	384
GhFAD2–3D	FAD2–4	Gh_D11G3169	D11:64332280..64333434	AY279314	1155	384
GhFAD2–4A	**/**	Gh_A01G2091	scaffold111_A01:182694..183845	/	1152	383
GhFAD2–4D	**/**	Gh_D01G1226	D01:30279978..30281129	/	1152	383

#### Expression pattern of different members of the GhFAD2 gene family

**Fig. 1:**
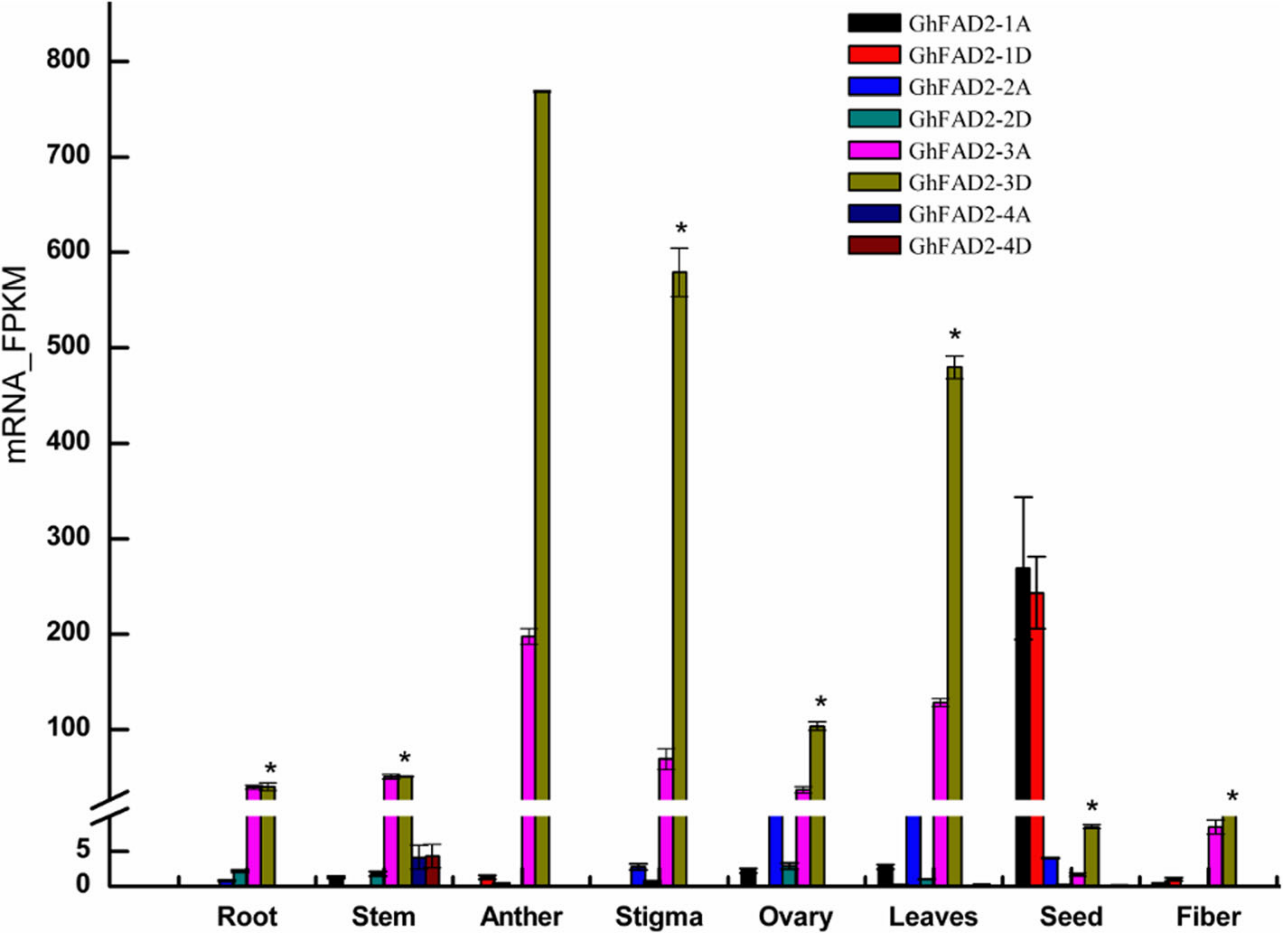
The expression profiles of *GhFAD2* genes in different tissues and at different developmental stages of cotton seed and fiber. Total RNA was isolated from root, leaves, stem, anther, stigma, ovary, seed and fiber. Each sample had three biological replicates. The seeds at 40 DPA and fiber at 24 DPA were used: In seeds, the expression level of *GhFAD2* reached its peak at 40 DPA [12]. It has been difficult to identify changes in gene expression for fiber after 30 DPA due to hard to extract mRNA. For each sample, a total of 3 μg RNA was used in preparing the RNA-seq library. Barcoded multiplexed RNA-seq libraries were created using the NEBNext® Ultra™ RNA Library Prep Kit for Illumina® (NEB, USA) according to the manufacturer’s protocol. Clean paired-end reads were aligned to the TM-1 reference genome, and the number of reads aligned to each gene was measured using HTSeq v0.6.1.The expression levels of individual genes were quantified using FPKM. Error bars are standard errors. Genes with an adjusted *P*-value < 0.05 found by DESeq were assigned as differentially expressed. * indicates a differentially expression of *GhFAD2–3D* between anther and other tissues (*P*adj < 0.05)

We analyzed the expression levels of each *GhFAD2* gene in different tissues and at different developmental stages of seeds and fibers using RNA-seq (Fig. 1). *GhFAD2–1A* and *GhFAD2–1D* were mainly expressed in developing seeds, particularly in 20–40 days post anthesis (DPA) seeds, expressed at very low levels in anther and ovary, and barely detectable in vegetative tissues and developing fibers, suggesting that the major role of *GhFAD2–1* is responsible for C18:2 biosynthesis in seeds, consistent with previous results [7, 38]. The expression of *GhFAD2–2*, particularly *GhFAD2–2A,* was mainly observed in ovaries and leaves and was very low or undetectable in other tissues. *GhFAD2–3* was constitutively highly expressed in all tissues, with a relatively low expression level in 40–60 DPA seeds. The two homoeologous *GhFAD2–3* sequences were equally expressed in most tissues but were significantly biased in anther, stigma and leaves, in which the expression level of *GhFAD2–3D* was much higher than that of *GhFAD2–3A*. For *GhFAD2–4*, there was very little expression in other tissues apart from stem. These results indicate that different *GhFAD2* genes are preferentially expressed in different tissues, and in some tissues the two homoeologs of the same *GhFAD2* gene are differentially expressed, pointing to a potential different function of the *GhFAD2* genes in cotton development. A very high expression level of *GhFAD2–3* in anthers suggests that lipid desaturation catalyzed by *GhFAD2–3* may have very important roles in anther development.

#### Molecular characterization of transgenic plants

A 517-bp fragment targeting both homoeologous *GhFAD2–3* sequences was used in making the hairpin construct. In total, 12 independent transgenic cotton lines were obtained by *Agrobacterium*-mediated transformation (Additional file 1: Figure S2). The transgenic cotton lines with the fusion fragment (1113 bp) from *GhFAD2–3* and the *gus* linker were considered as positive transgenic events. To investigate the effect of downregulation of *GhFAD2–3* on changes in the anther transcriptome, we compared transcriptomes of wild-type and *fad2–3* using RNAs from anthers of the meiosis and tetrad stages, considering that the differences in cellular phenotypes between the wild-type and *fad2–3* anthers began to be observed at the meiotic stage by transmission electron microscopy (see below). As expected, both homoeologous *GhFAD2–3* (*Gh_D11G3169* and *Gh_A11G2814*) were downregulated at both stages, although downregulation at the tetrad stage was more statistically significant (Additional file 1: Table S1). Among the other *GhFAD2* genes, *GhFAD2–1* (*Gh_A13G1850* and *Gh_D13G2238*) was significantly downregulated at both stages in *fad2–3* due to its closest sequence similarity with *GhFAD2–3*. However, compared to *GhFAD2–3, GhFAD2–2D* and *GhFAD2–4A* had a negligent expression level in anthers, and could thus hardly have function in anther development.

Significantly changed genes encoding key enzymes of these pathways were shown in Additional file 1: Table S1. These results revealed that many significantly changed pathways are related to the metabolism of lipids and their derivers; among them were those involved in biosynthesis of unsaturated fatty acids, alpha-linolenic acid metabolism, and the biosynthesis of cutin, suberin and wax. Apart from *GhFAD2*, DEGs related to biosynthesis of unsaturated fatty acids also included *GhFAD3* (such as *Gh_A09G0848* and *Gh_A07G0946*). The expression levels of genes involved in linoleic acid and alpha-linolenic acid metabolism was also statistically significant at the meiosis stage or tetrad stage in the *fad2–3* anthers. In addition, fatty acyl-CoA reductase (FAR) catalyzes the reduction of fatty acyl-CoA to fatty alcohols, which are essential components of wax and cutin monomers. *GhFAR2* genes (*Gh_A09G1215*), orthologous to *Arabidopsis MS2* and rice *DPW* that are related to wax biosynthesis, were found to be downregulated at the tetrad stage in *fad2–3*. *GhCYP86B1* (*Gh_D04G1447*, *Gh_A04G0930*, *Gh_A03G2129* and *Gh_D02G1587*), a very long chain fatty acid hydroxylase specifically involved in cutin and suberin biosynthesis, was significantly upregulated in the *fad2–3* anther. Furthermore, Cytochrome P450 *CYP704B1*, which participates in catalyzing omega-hydroxylation of long-chain fatty acids was also differentially expressed. Significant differences were also observed in the expression levels of some genes, such as those encoding peroxygenase and aldehyde dehydrogenase.

#### Phenotypic analysis of the RNAi plants

**Fig. 2:**
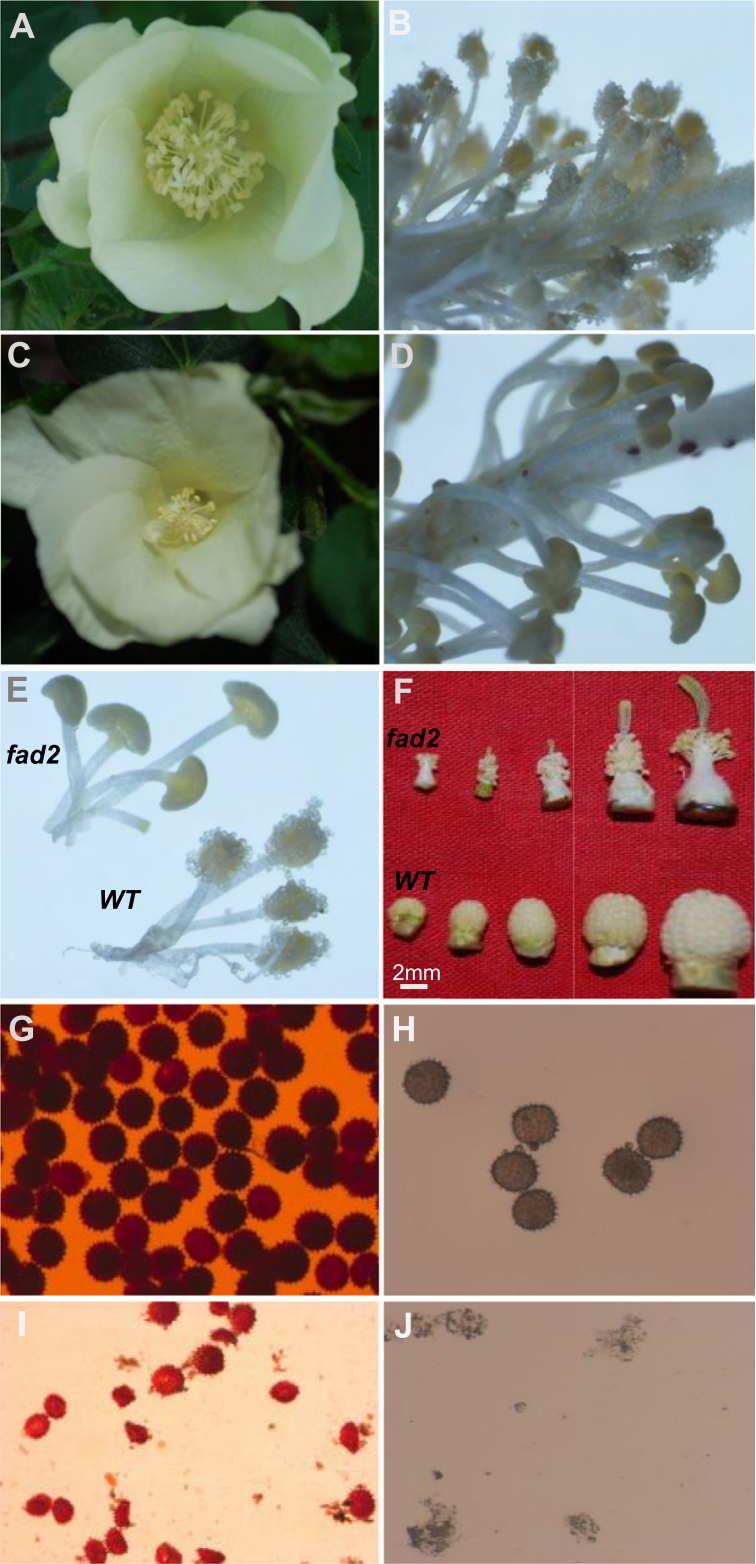
Comparison of pollen phenotype and vitality between the wild-type (WT) and the *fad2–3* plants. **a**. Wild-type flower; **b**. Wild-type anthers; **c**. *fad2–3* flower. The magnification was 5 times. **d**. *fad2–3* anthers. The magnification was 5 times. **e**. Comparison of wild-type and *fad2–3* mature anthers. The magnification was 5 times. **f**. Comparison of developing anthers from the wild-type and the *fad2–3* plants; shown are de-bracted young buds collected every 4 days after bud emergence; **g**. Wild-type pollen grains stained by I_2_-KI. The magnification was 50 times. **h**. Wild-type pollens after acetolysis treatment. The magnification was 50 times. **i**. *fad2–3* pollen grains stained by I_2_-KI. The magnification was 50 times. **j**. *fad2–3* pollen after acetolysis treatment. The magnification was 50 times

**Fig. 3:**
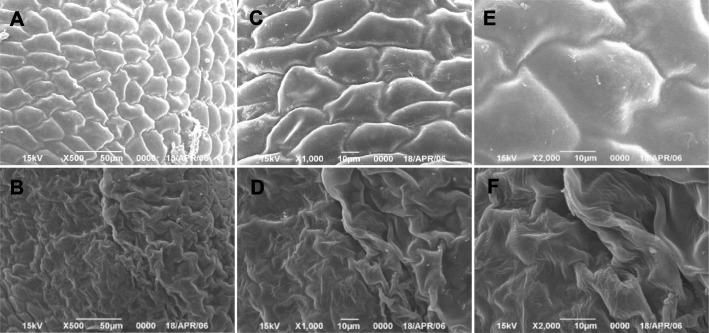
Scanning electron microscopy of the mature anthers from the wild-type and the *fad2–3* plants. **a**, **c**, and **e**. mature wild-type anthers. The magnification was 500, 1000 and 2000 times, respectively. **b**, **d**, and **f**. mature *fad2–3* anther. The magnification was 500, 1000 and 2000 times, respectively

All *fad2–3* produced lots of completely sterile flowers, although they were able to open as fully as the wild-type flowers. At anthesis, wild-type anthers dehisced to release pollen grains for pollination, whereas *fad2–3* anthers did not dehisce, had a smooth and shiny epidermal surface and were plate-shaped (Fig. 2). In wild-type, anthers were clustered in fascicles, and stamens wrapped up stigma before pollen maturation. The *fad2–3* had fewer anthers than wild-type, and its anthers did not wrap up stigma as did the wild-type anthers. As a result, stigma of *fad2–3* stood out in the anther cluster. We also manually opened *fad2–3* anthers and compared its pollen grains with those of wild-type. It was obvious that *fad2–3* had fewer pollen grains. While the mature pollen grains of wild-type were spherical and became dark brown when treated with I_2_-KI (Fig. 2), the manually released *fad2–3* pollen grains were smaller, shrunken, irregularly shaped, and were yellow brown when stained by I_2_-KI (Fig. 2). After acetolysis treatment, wild-type pollen grains remained intact, whereas *fad2–3* pollens were severely damaged and became transparent (Fig. 2), suggesting that *fad2–3* pollens were sensitive to acetolysis probably due to lack of sporopollenin in the outer pollen wall; i.e.*,* the exine. We compared the surface structure of wild-type and *fad2–3* anthers harvested from 1 day before anthesis using scanning electron microscopy (Fig. 3). Compared with the well-formed, relatively smooth wild-type anthers, *fad2–3* anthers had a severely shrunken, atrophied and disfigured outer surface.

**Fig. 4:**
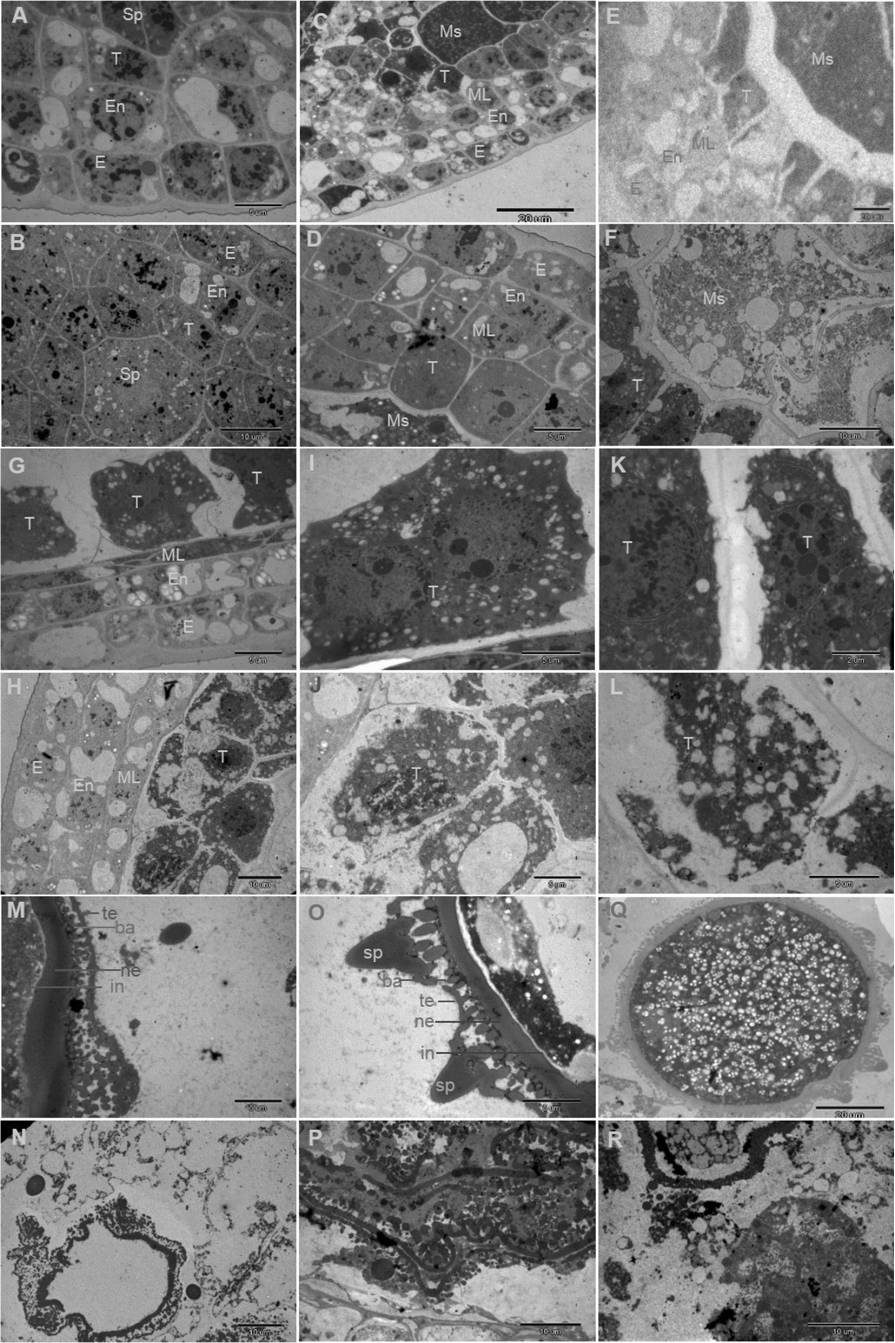
Transmission electron micrographs of anthers from the wild-type and the *fad2–3* plants. A, C, E, G, I, K, M, O and Q, wild-type anthers at different developmental stages. B, D, F, H, J, L, N, P and R, *fad2–3* anthers at different developmental stages. (**a**) Wild-type anthers with sporogenous cells and three layers of parietal cells. Bars = 5 μm. (**b**) *fad2–3* anthers with sporogenous cells and three layers of parietal cells. Bars = 10 μm. (**c**) Wild-type anthers with microsporocytes and four layers of parietal cells. Bars = 20 μm. (**d**) *fad2–3* anthers with microsporocytes and four layers of parietal cells. Bars = 5 μm. (**e**) Wild-type anthers at the meiosis stage. Bars = 20 μm. (**f**) *fad2–3* anthers at the meiosis stage. Bars = 10 μm. (**g**) Wild-type anthers at the early tetrad stage. Bars = 5 μm. (**h**) *fad2–3* anthers at the early tetrad stage. Bars = 10 μm. (**i**) High magnification of the wild-type tapetum showing double nuclei at the tetrad stage. Bars = 5 μm. (**j**) High magnification of the *fad2–3* tapetum at the tetrad stage. Bars = 5 μm. (**k**) High magnification of the wild-type tapetum showing mitochondrion and endoplasmic reticulum. Bars = 2 μm. (**l**) High magnification of the *fad2–3* tapetum showing large vacuoles, defective plastids. Bars = 2 μm. (**m**) Wild-type microspore at the middle developmental stage; tectum, bacula and nexine appeared. Bars = 5 μm. (**n**) *fad2–3* microspore at the middle developmental stage; bacula and nexine appeared and showed abnormalities. Bars = 5 μm. (**o**) The spinules protruding from the wild-type microspore exine were formed at stages of mitosis. Bars = 5 μm. (**p**) *fad2–3* microspore exine showing obvious abnormality. Bars = 5 μm. (**q**) Wild-type mature pollen grains were uniformly distributed in the small vacuole. Bars = 20 μm. (**r**) The degraded *fad2–3* anthers exhibiting crushed cell structure. E, epidermis; En, endothecium; ML, middle layer; Sp, sporogenous cells; Ms., microsporocyte; T, tapetum; te, tectum; ne, nexine; ba, bacula; in,intine; sp., spinules

To have a deeper understanding of the abnormalities of the *fad2–3* anther development, we collected anthers at different developmental stages from *fad2–3* and wild-type, and analyzed anther cross sections by transmission electron microscopy. Before the sporogenous cell stage, in both *fad2–3* and wild-type, stamen primordia were initiated normally and large archesporial cells could be detected in developing anthers. Wild-type and *fad2–3* anthers had similar cytological characteristics. At the sporogenous cell stage, wild-type and *fad2–3* anthers had no obvious differences in cellular structures (Fig. 4a and b). At the microsporocyte stage, the four layers of the anther wall; i.e.*,* epidermis, endothecium, middle layer and tapetum were well differentiated (Fig. 4c and d) in both wild-type and *fad2–3*. A clear defect in the *fad2–3* anther was first observed at the meiosis stage. Compared with the wild-type anther, the *fad2–3* anther showed cytoplasmic diffusion in microsporocytes and disintegration of the tapetum at this stage (Fig. 4e and f). At the early tetrad stage, the middle layer cells of the wild-type anther became narrow and deformed and began to show signs of degeneration (Fig. 4g). The tapetum cells were large, had thick cytoplasm rich in endoplasmic reticulum, mitochondria and plastids, and often contained double nuclei. Profuse vesicles with dense electron substances were continuously produced by the endoplasmic reticulum (Fig. 4i). Later, the endoplasmic reticulum of tapetum disappeared, resulting in accumulation of orbicules, polyvesiculate bodies and lipid bodies in the tapetum, and finally degradation of the tapetum. In contrast, the middle layer cells of the *fad2–3* anther did not become thinner at the early tetrad stage (Fig. 4H). However, the tapetal cells of the *fad2–3* anther showed significant abnormalities, including having many large vacuoles and defective plastids, without obvious accumulation of lipid droplets in elaioplasts and absence of dual nuclei (Fig. 4j). There were large numbers of vacuoles and dilated vesicles of endoplasmic reticulum in the tapetum cells (Fig. 4l). With the development of microspores, formation of primexine followed by bacula, tectum and nexine could be seen in the wild-type anther (Fig. 4m). Later, microspore exine and intine were fully thickened, and spinules protruding from the exine were formed (Fig. 4m and o). Further, vacuolization was observed in mononucleate-free microspores (Fig. 4q). In the *fad2–3* anther, although bacula could form normally with its upper and lower ends extended laterally during the development of pollen exine, the development of microspore exine showed obvious abnormalities, including being unable to form tectum uniformly composed of small spinules (Fig. 4n and p), shrunken protoplasts showing breakage of the cell membrane, and external flow of cytoplasm. Other abnormalities were a concentration of partial cytoplasm in the center of the microspore, dissolution of nuclear membranes, and disintegration of the nucleus and cytoplasm (Fig. 4r).

#### Silencing of GhFAD2–3 decreased C18:2 content in anthers

**Fig. 5:**
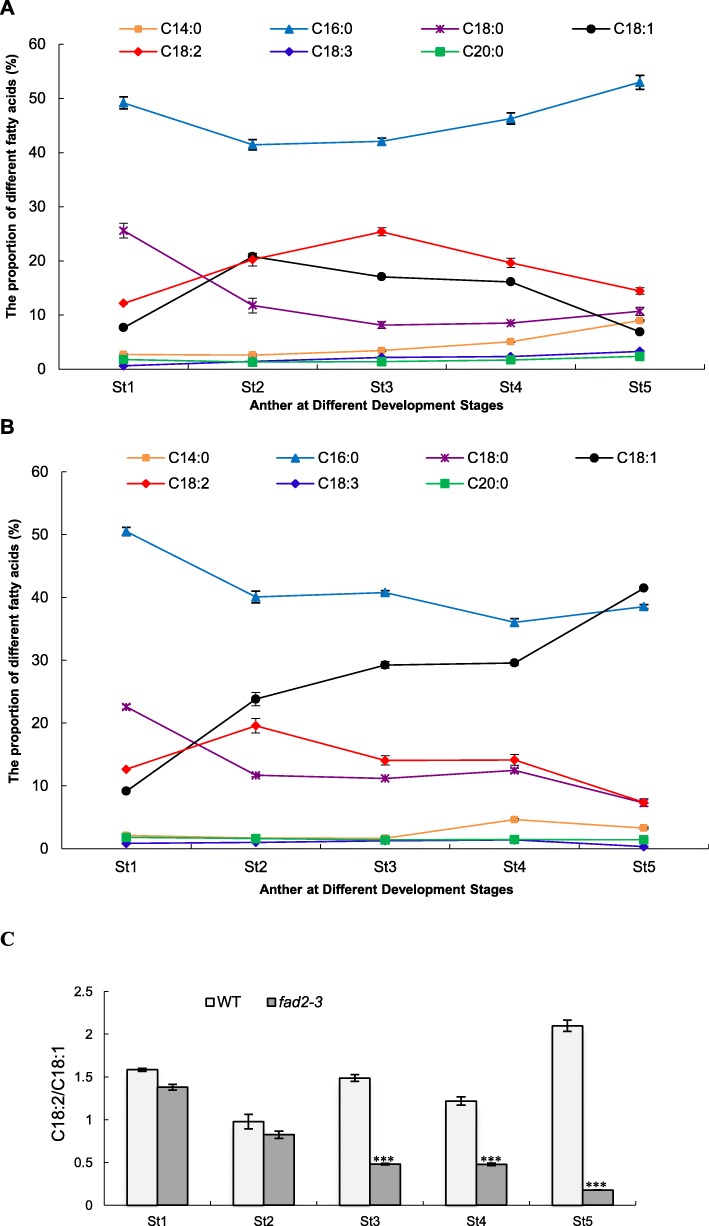
The proportion of fatty acid compositions in anthers at different developmental stages. **a**, wild-type anthers; **b**, *fad2–3* anthers; **c**, the ratio of C18:2 to C18:1. Anthers at different developmental stages was used for fatty acid assay. St1-St5: Anther at different developmental stages; St1, Sporogenous cell stage; St2, Microsporocyte stage; St3, Meiosis stage; St4, Tetrad stage; St5, Pollen maturation stage. The fatty acid methyl esters were prepared by alkaline transmethylation. The analyses were performed using GCMS-QP2020 at an electron ionization of 70 eV with an HP-88 column. The quantification was carried out according to the response value of quantitative ions and the established standard curve. Each test was repeated three times, and the content of each fatty acid composition was calculated as the percentage of total measured fatty acids. The ratio of C18:2/C18:1 is calculated by dividing the relative percentage content of C18:2 from that of C18:1 at the same developmental stage. Each bar represents the mean data of three biological replicates. Error bars are standard errors. Asterisks denote significant differences to wild-type (WT) as determined by Student’s *t* test: ***p < 0.001

The main fatty acids in cotton anthers are saturated myristic acid (C14:0), palmitic acid (C16:0), stearic acid (C18:0) and arachidic acid (C20:0), monounsaturated oleic acid (C18:1), and polyunsaturated linoleic acid (C18:2) and α-linolenic acid (C18:3). In the wild-type anther, C16:0 was the main fatty acid and reached the maximum in the mature pollen (Fig. 5a). C18:0 accumulated mainly at the early stage of anther development and its relative content significantly decreased from 25.56% at the sporogenous cell stage to 8.14% at the meiosis stage and slightly increased again at the pollen maturation stage. The relative content of C14:0, C20:0 and C18:3 was relatively low in all stages of anthers, although C14:0 was increased up to 8.97% at the mature pollen stage. C18:1 and C18:2 had a similar dynamitic change pattern during anther development and reached their maximum at the microsporocyte and meiosis stage, respectively, suggesting that biogenesis of C18:2 largely depends on the amount of C18:1, which may be negatively correlated with that of C16:0.

In the *fad2–3* anthers, due to the significantly reduced level of *GhFAD2–3*, conversion of C18:1 into C18:2 was compromised after the microsporocyte stage. Consequently, a significant reduction in C18:2 was observed at the meiosis and tetrad stages, while the relative content of C18:1 was significantly increased at these two stages (Fig. 5b). Significant reduction in C18:2 at around the meiosis stage coincides with the developmental abnormalities observed at this stage of anther development in *fad2–3*. A reduction (49.18%) in C18:2 was detected in the mature anthers of *fad2–3* compared to that of wild-type, but C18:1 increased almost five-fold in *fad2–3* compared to wild-type, suggesting that the biosynthesis steps from C16:0 to C18:1 were not greatly affected by silencing of *GhFAD2–3*, resulting in accumulation of C18:1.

The ratio of C18:2/C18:1 was analyzed during anther development. The results showed that there was no significant difference between *fad2–3* and WT during early anther development (Fig. 5c). At the meiosis stage, the ratio of C18:2/C18:1 was 1.49 in WT anthers. However, the corresponding ratio was only 0.48 in *fad2–3* anthers. Compared with the WT anther, there was a more significant decrease in the ratio of C18:2/C18:1 in the *fad2–3* anther at the pollen maturation stage (Fig. 5c).

#### Cuticular wax constituents and cutin monomer of cotton anthers

**Fig. 6:**
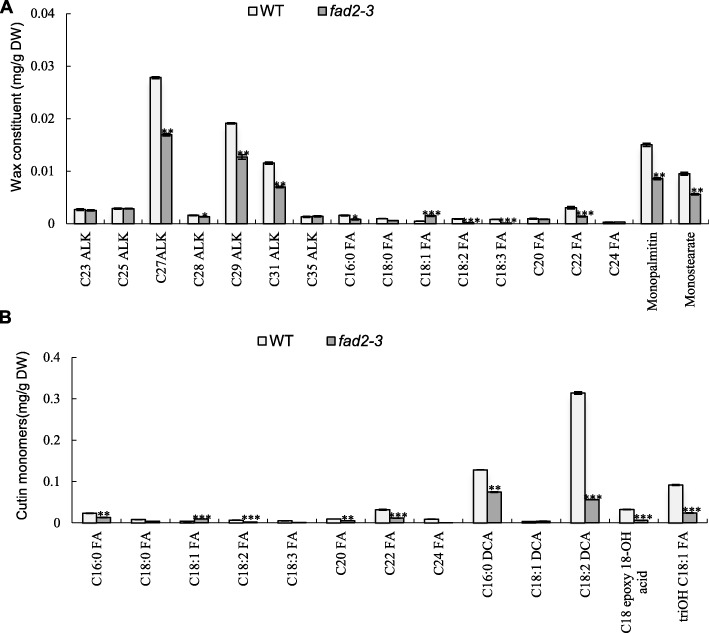
Analysis of anther wax and cutin in the wild type and *fad2–3.* (**a**) Wax constituents in the wild-type and *fad2–3.* (**b**) Cutin monomers in the wild-type and *fad2–3*. C23 ALK, tricosane; C25 ALK, pentacosane; C27 ALK, heptacosane; C28 ALK, octacosane; C29 ALK, nonacosane; C31 ALK, hentriacontane; C35 ALK, pentatriacontane. C16:0 FA, hexadecanoic acid; C18:0 FA, octadecanoic acid; C18:1 FA, 9-octadecenoic acid; C18:2 FA, 9,12-octadecadienoic acid; C18:3 FA, 9,12,15-octadecatrienoic acid; C20 FA, eicosanoic acid; C22 FA, docosanoic acid; C24 FA, tetracosanoic. C16:0 DCA, hexadecane-1,16-dioic acid; C18:1 DCA, α, ω-octadecenoic acid; C18:2 DCA, α, ω-octadecadiendioic acid; triOH C18:1 FA, 9,10,18-trihydroxy octadecenoic acid; 9,10 Epoxy 18-OH acid, 9,10-epoxy-18-OH-C18:1; DW, dry weight. The wax of anther at mature pollen stage was analyzed according to Jung et al. [26]. The wax monomer was derivatized with 1 ml BFTSA in 1 ml pyridine (1:1) for 40 min at 70 °C before GC-MS analysis. The constituent analyses were performed using GCMS-QP2020 with a DB-1 column. Each compound was quantified against the internal standard by automatic integrating the peak areas. The protocol for lipid polyester analysis was performed according to Li-Beisson et al. [50]. The cutin monomer fraction was derivatized with BFTSA/pyridine (1:1) for 60 min at 70 °C. The constituent were analyzed using GCMS-QP2020 with a DB-1 column. The GC-MS was conducted according to Li-Beisson et al. [50] with helium carrier gas at 2 ml/min. Each compound was quantified on the basis of their total ion current as described by Li-Beisson et al. [50]. Error bars are standard errors. Values represent the means ± SE, *n* = 3. Asterisks denote significant differences to wild-type (WT) as determined by Student’s *t* test: ***p < 0.001; **p < 0.01; *p < 0.05

To eliminate possible effects caused by the changed *fad2–3* anther morphology, the experiments were performed using anthers harvested from 1 day before anthesis. The cuticular waxes and cutin of mature anthers was extracted and their components were analyzed accordingly by GC-MS (Fig. 6a).

Interestingly, in wild-type anthers, wax constituents contain wax esters at high content (over 50%), prominently monopalmitin and monostearate, which are products of the acyl reduction pathway. This is different from waxy components in anthers of rice and maize [31, 39], perhaps because cotton is a woody perennial plant, and it seems to be similar to that in wax constituents of Jojoba [40]. The content of alkanes was approximately 40%, mainly with chain lengths ranging from 23 to 35 carbons. Compared with the wild-type, there was no significant difference in the major components of wax in *fad2–3*. Although the content of fatty acid components was relatively low in wax constituents, *fad2–3* had significantly lower levels of C18:2 and C18:3, but a relatively higher level of C18:1. The decrease in C16:0 and C18:0 was also observed in the anther wax of *fad2–3*. These results indicated that C18:2 and C18:3 were also important components of plant epidermis and downregulation of *GhFAD2–3* affected the relative content of components in the cuticular layer.

Cutin monomers in the cuticular layer were methylated by methanolic HCl and reanalyzed by GC-MS. The major monomer was α, ω-octadecadiendioic acid (C18:2 DCA), which is a usual constituent of cutin. 9,10,18-trihydroxy octadecenoic acid (9,10,18-triOH C18:1 FA), hexadecane-1,16-dioic acid (C16:2 DCA) and 9,10-epoxy-18-OH-C18:1 (9,10 Epoxy 18-OH Acid) at relatively high levels had also been detected by GC-MS in wild-type anthers (Fig. 6b). Cutin monomers also include fatty acid components such as C18:2 and C18:3. The strongest effects of the *fad2–3* anther on cutin monomers were apparent in the unsaturated C18:2 DCA and 9,10,18-triOH C18:1FA. Especially, the C18:2 DCA content decreased by 82.16% in *fad2–3*. Our results were also consistent with previous reports that the *Arabidopsis fad2* mutant showed a decrease in double unsaturated C18 α, ω-diacids in leaf polyester [41]. Nevertheless, C18:2 and C18:3 are important substrates for biosynthesis of many other lipids that are essential structural components of anthers and the pollen wall.

## Discussion

### Functional specificity and redundancy of *GhFAD2* genes

Each individual *GhFAD2* gene is expected to function as a desaturase to convert C18:1 into C18:2 in different cotton organs and/or tissues, a process that is presumably determined by the expression specificity and level of each *GhFAD2*. Among the four pairs of *GhFAD2* genes, *GhFAD2–2* and *GhFAD2–4* were expressed at very low levels in the tissues analyzed in this study, suggesting a limited role or a specific role in the tissues not analyzed in this study of these genes in cotton development. *GhFAD2–1* seems to be specifically expressed in developing seeds, with the highest expression level detected in the 40 DPA seeds (Fig. 1), consistent with its role in the accumulation of C18:2 in seeds [7]. In cotton seeds, the relative content of C18:2 could reach over 50% of the total fatty acid content [12]. In contrast, *GhFAD2–3* seems to be ubiquitously expressed in all tissues analyzed, but its expression level was remarkably lower than that of *GhFAD2–1* in the 40 DPA seeds although its expression levels in the 5 DPA and 20 DPA seeds were significantly higher than or similar to that of *GhFAD2–1*, respectively (Fig. 1). The highest expression level of *GhFAD2–3*, particularly *GhFAD2–3D*, was observed in anthers, whereas the other three pairs of *GhFAD2* genes were not or expressed at very low levels in anther. This expression pattern suggests that *GhFAD2–3* is the major, if not the sole, gene responsible for the synthesis of C18:2 in anthers and is important for anther development. The male sterile phenotype observed in *fad2–3* supported this conclusion. *GhFAD2–3*, particularly *GhFAD2–3D*, was also relatively highly expressed in stigma and leaves, but we did not observe obvious phenotypic changes in these two organs in *fad2–3*, probably due to the presence of a functional *GhFAD2–2* that was not a target of the 517-bp fragment used in generation of *fad2–3*. This observation suggests that different *GhFAD2* genes may be functionally redundant.

One interesting observation was the significantly biased expression levels of the two homoeologous *GhFAD2–3* in anthers, stigma and leaves (Fig. 1). The significantly higher expression level of *GhFAD2–3D* would suggest it is the major functional gene. This speculation could not be tested by using *fad2–3* generated in this study because both gene homeologs are targets of the RNAi construct but can be tested by gene homolog-specific knock-out using the gene editing approach.

### A role for *GhFAD2–3* in anther and pollen development

Fatty acid metabolism is an essential physiological process throughout the plant life cycle. In higher plants, acetyl-CoA carboxylase carboxylates acetyl-CoA to form malonyl-CoA, which is further converted by fatty-acid synthase (FAS) to long-chain fatty acids via the six recurring reactions, until the C16:0 is produced. When the 16:0 carbon fatty acids (FAs) are formed, it then undergoes some modifications leading to desaturation and/or elongation. The elongation begins with stearate (C18:0) and is mainly performed by several membrane-bound enzymes in the endoplasmic reticulum (ER). C18:0 was also further dehydrogenated by Δ^9^-stearyl-ACP desaturase (SAD) to form monounsaturated C18:1. After that, most PUFAs are synthesized by desaturases located in the ER, namely, FAD2 (C18:1 to C18:2 desaturation) and FAD3 (C18:2 to C18:3 desaturation). Most cuticular wax and cutin are derived from fatty acid precursors and play important roles in developmental events and physiological functions. Our results also showed that the major wax constituents in the cotton anthers were wax esters and alkanes. Importantly, it had been reported that fatty acid desaturases, including FAD2, are responsible for the biosynthesis of 30–35% of the cutin monomers from unsaturated C18 aliphatics [42]. Similar to the observation of a decrease in the double unsaturated C18 diacids in leaf polyester of the *Arabidopsis fad2* mutant [41], we showed that the contents of polyunsaturated C18 dioic acid were significantly reduced in the anther cutin of *fad2–3*, suggesting that maintaining a certain level of polyunsaturated C18 is important for proper development of the cuticular structure of cotton anthers.

**Fig. 7:**
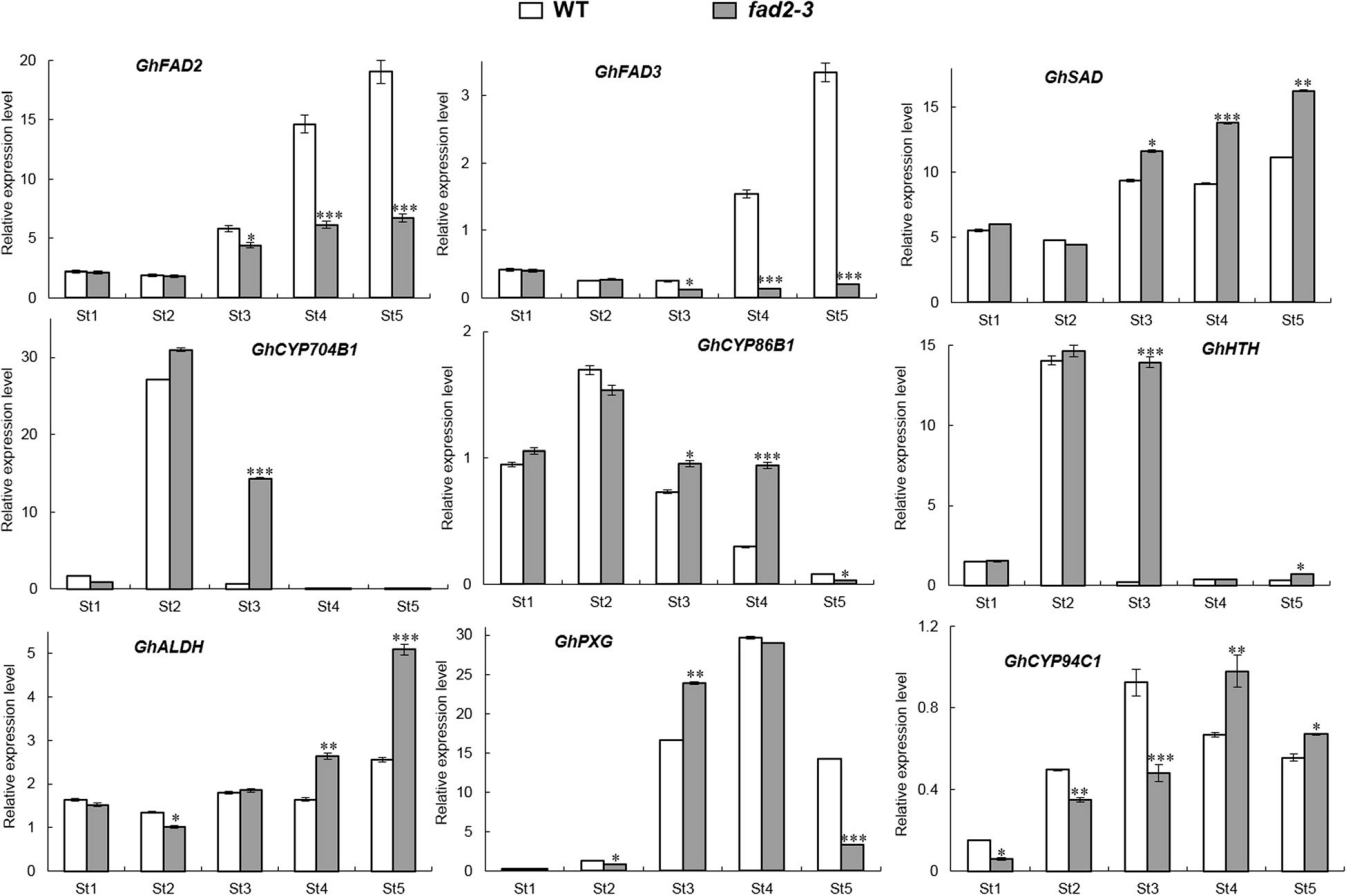
qRT-PCR analysis of some candidate genes involved in the biosynthetic pathways for cutin monomers in in wild-type and *fad2–3.* St1-St5: Anther at different developmental stages; St1, Sporogenous cell stage; St2, Microsporocyte stage; St3, Meiosis stage; St4, Tetrad stage; St5, Pollen maturation stage. The *GhFAD2–1, GhFAD3, GhSAD, GhCYP704B1, GhCYP86B1, GhHTH, GhALDH, GhPXG,* and *GhCYP94C1* mRNA abundance was determined by qRT-PCR, respectively. These genes encodes enzymes indicated as follows: Fatty acid desaturase 2, Fatty acid desaturase 3, Δ^9^-stearyl-ACP desaturase, Cytochrome P450 704B1, Cytochrome P450 86B1, ω-hydroxyacid dehydrogenase, aldehyde dehydrogenase, peroxygenase and Cytochrome P450 94C1. *GhUBQ14* was used as a reference gene. All qRT–PCR reactions were performed in triplicate. Relative gene expression levels of target genes were normalized against Ct values for *GhUBQ14*, and the fold change (2^–ΔΔCt^) was determined by comparison to average expression levels. Significant differences from control were marked with * (*P < 0.05*), ** (*P < 0.01*) and *** (*P < 0.001*)

**Fig. 8:**
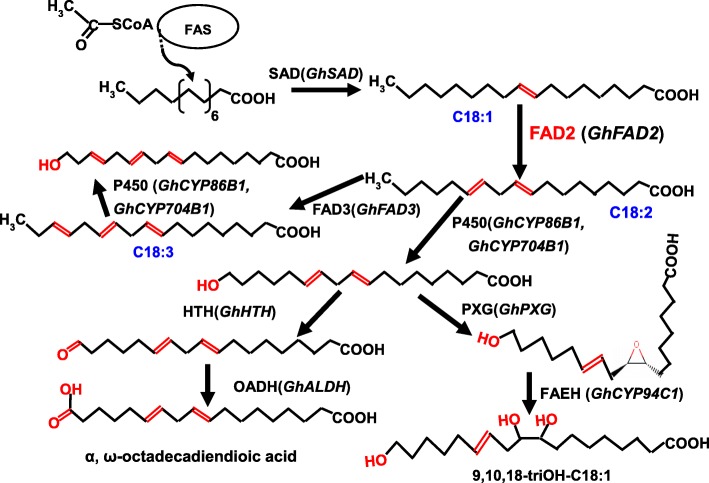
Model of FAD2 involved in the primary pathways for cutin monomers synthesis in *Gossypium*. *Gossypium* candidate genes are given in parenthesis. Arrow thickness indicates the extend of carbon flux. Enzymes presumably involved are indicated as follows: FAS, fatty acid synthase; FAD2, fatty acid desaturase 2; FAD3, fatty acid desaturase 3; SAD, stearoyl-ACP desaturase; P450, Cytochrome P450 monooxygenase; HTH, ω-hydroxyacid dehydrogenase; OADH, ω-oxo-acid dehydrogenase (aldehyde dehydrogenase); PXG, peroxygenase; FAEH, fatty acid epoxide hydrolase

In our study, silencing *GhFAD2–3* induced transcriptional changes during anther development (Additional file 1: Table. S1). Our qRT-PCR results were in accordance with transcriptional analysis (Fig. 7), as silencing of *GhFAD2–3* in anthers resulted in changing expression levels of many genes, such as *GhCYP86B1*, *GhCYP704B1* and *GhCYP94C1*. With our study, the probable scheme of FAD2 involved in the primary pathways for cutin monomers synthesis in *Gossypium* was proposed (Fig. 8). In this pathway, the ω-hydroxylation reaction is typically catalyzed by cytochrome P450 monooxygenases, particularly of the *GhCYP86B1* and *GhCYP704B1*. The ω-hydroxy FAs could be further oxidized by ω-hydroxyacid dehydrogenase (HTH) to ω-oxo FAs. *GhALDH* encodes an aldehyde dehydrogenase that further catalyzes ω-oxo FAs to produce α, ω-dicarboxylic FAs. On the other hand, peroxygenase (PXG) catalyzes the hydroperoxide-dependent epoxidation of unsaturated fatty acids, and then *GhCYP94C1* with high omega-hydroxylase activity to 9, 10-epoxyoleic acid metabolized C18 unsaturated FAs to produce polyhydroxy-octadecenoic acid. Mutation in any gene encoding the enzymes involved in the pathway could cause a lack of synthesis of cutin/wax and sporopollenin precursors, resulting in failure to form normal pollen exine and anther cuticle that had also been reported [18, 23, 33].

In our model, *GhFAD2* could play an important role in the biosynthesis of the cutin and suberin monomers. The *fad2–3* is deficient in its ability to catalyze C18:1 to C18:2, which finally results in anther polyesters with less α, ω-octadecadiendioic acid (C_18:2_) and polyhydroxy-octadecenoic acid (9,10,18-triOH C18:1 FA). Our qRT-PCR results showed that the expression of *GhFAD2* was relatively low at the sporogenous cell stage and microsporocyte stage, significantly increased at the tetrad stage, and peaked at the pollen maturation stage in the control plants (Fig. 7). In *fad2–3*, *GhFAD2* had a very similar dynamic expression change at different anther developmental stages; however, its expression level was significantly lower than that of the wild-type at the time points investigated, particularly from the tetrad stage to the pollen maturation stage (Fig. 7). *GhFAD3* also had a similar expression change in the wild-type and *fad2–3* during anther development. Correspondingly, from the sporogenous cell stage to the microsporocyte stage, there was no difference in the relative content of C18:2 and C18:3 between the wild-type and *fad2–3* anthers, but from the microsporocyte stage until the mature pollen stage, the relative content of C18:2 and C18:3 was significantly lower in *fad2–3* than in wild-type (Fig. 5).

Overall, in *fad2–3*, the expression of *GhFAD2* and *GhFAD3* genes was significantly inhibited; however, the expression levels of the above determined related genes in the pathway, such as *GhSAD*, *GhCYP86B1, GhCYP94C1, GhHTH* and *GhALDH*, were apparently upregulated, especially at the meiosis stage and/or tetrad stage. Thus, it is interesting that the upregulation of the expression of these genes in *fad2–3* was accompanied by the increase in C18:1 content relative to the control anther. Similar phenomena have also been previously observed with transgene expression of *FAD2*, which produces unusual FAs, including epoxidation, hydroxylation and double bonding conjugation [40, 41]. One hypothesis explaining this phenotype is that unusual FA products inhibit the activity of FAD2, thereby effectively preventing the conversion of C18:1 to C18:2 [43–46]. In cottonseed, the relative content of C18:2 peaked in the mature seeds, accounting for over 50% of the total fatty acid content of seed [12]. However, the accumulated unusual FA products, such as epoxy fatty acid, hydroxyl acid, and dioic acid, may act as inhibitors of *GhFAD2* expression in anthers. Thus, this regulatory mechanism may account for part of why the relative proportion of C18:2 is not very high in anthers. In *fad2–3*, the expression of *GhSAD* was significantly upregulated at the meiosis stage and the tetrad stage, which would further lead to a much higher proportion of C18:1 content. Under this metabolic scenario, *GhFAD2* would be further inhibited by the increased expression of related genes in the pathway, such as *GhCYP86B1*, *GhALDH* and *GhCYP94C1*, resulting in relatively low C18:2 content at the meiosis stage and the tetrad stage in *fad2–3*. In contrast, the expression of related genes in the pathway to synthesize waxy and cutin monomers could be induced by a low level of C18:2 content. This may be a self-protective mechanism of plant cells. Compared to wild type, *GhHTH* and *GhCYP704B1* shows significantly higher expression level at the meiosis stage. However, this does not mean that it could result in an increase in the content of hydroxy and epoxy fatty acids. At the tetrad stage, the expression of *GhHTH* and *GhCYP704B1* was very low in both wild type and *fad2–3*, and there was no significant difference between them. The anthers of *fad2–3* were smaller than those of wild-type, and the *fad2–3* pollen grains appeared to lack the exine layer. These results suggest that the lack of C18:2 and C18:3 have adverse effects on the establishment of functional anther cuticles and pollen exine in *fad2–3*, probably a result of insufficient biosynthesis and/or deposits of sporopollenin in these protective walls, as suggested by the observation that the *fad2–3* pollen grains were unresistant to acetolysis treatment. The high C18:1 content in cottonseeds by specific inhibition of the expression of *GhFAD2–1* had disadvantageous effects on seed vigor [12]. As in cottonseed, it seemed to suggest that the too high accumulation of C18:1 was also unfavorable to the development of anthers.

In plants, the synthesis of various fatty acid components has a complex interrelationship, and the synthesis of other fatty acids will be affected when the synthesis of a particular fatty acid component is regulated. In general, with the increase in C18:1 content, the content of C16:1 decreased in this study. The decrease in C16:0 was also observed in the anther wax of *fad2–3*. Accordingly, the content of C16:0 DCA was reduced in the anther cutin of *fad2–3*. The relative proportion of fatty acid components accumulated in plant tissues is very important for ensuring that they could preserve the basic physiological activities. Changing the relative content and proportion of one or some fatty acid components in plant tissues may adversely affect vegetative or reproductive growth of plants. It had been reported that the *Arabidopsis fad2* mutants showed dwarf phenotypes and the levels of PUFAs in phospholipids were relatively low. The limited membrane fluidity of the *fad2* mutant resulted in its inability to survive at a low temperature [47]. We had also previously found that the decreased C18:2 content in cottonseeds by specific inhibition of the expression of *GhFAD2–1* had disadvantageous effects on seed vigor [12]. C18:2 and C18:3 could serve as the essential structural components of anthers and pollen walls and/or as the substrates for biosynthesis of signaling molecules and/or hormones, such as Jasmonic acid (JA), essential for anther and pollen development. Jasmonic acid is a carbocyclic fatty acid and plays an essential role in pollen and anther development. Several *Arabidopsis* male sterile mutants have been shown to be caused by mutation in genes involved in JA biosynthesis and/or signaling pathways, such as *coi1* [48] and *opr3/dde1* [49, 50]. It has been reported that the crucial requirement for C18:3 in *A. thaliana* pollen development and anther dehiscence seems to be as a substrate for JA biosynthesis through the octadecanoid pathway [51]; however, the threshold requirement for C18:3 is very low, and anthers with only 1–2% of C18:3 could remain fertile. The wild-type cotton anthers contain a relatively low level of C18:3 compared to C18:1 and C18:2 but could have a level higher than the low threshold reported in *Arabidopsis*. The C18:3 content was significantly reduced in the *fad2–3* anthers and became as low as 0.3% (~ 10-fold reduction) at the mature pollen stage (Fig. 5). Taken together, C18:2 and C18:3 are essential structural components of anthers and are also important substrates for biosynthesis of many other lipids in anther development.

## Conclusion

In this study, we show functional specificity and redundancy of *GhFAD2* genes that would be helpful to understand gene expression and regulation in allopolyploid crops. We report for the first time on the dynamic changes in fatty acid constituents during anther development and showed that silencing of *GhFAD2–3* reduced PUFAs (mainly C18:2 and C18:3) in anthers, severely hampered pollen development and significantly reduced the number and viability of pollen grains. Our results demonstrated that the relative proportions of fatty acid components accumulated in plant tissues is very important to ensure that they could preserve the basic physiological activities. In the future, a comprehensive and in-depth study of unsaturated fatty acids and their derivatives in plant cells would help us to better control plant growth and development on the basis of understanding the physiological significance of the formation of plant fatty acid components.

## Methods

### Characterization of the *GhFAD2* gene family

The genome sequences and annotation files of *G. hirsutum* (AD1_NBI), *G. raimondii* (D5_JGI) and *G. arboreum* (A2_BGI) were downloaded from CottonGen (https://www.cottongen.org). To identify the members of the *GhFAD2* family genes, the amino acid sequence of the previously reported GhFAD2 (GenBank accession no. X97016) was used to search for its homologs in the annotated proteins of the three genomes using BLASTP (E-value ≤10^− 50^). The hits with all three conserved histidine-clusters observed in all reported plant FAD2 sequences were considered as GhFAD2. The relationship of the identified *GhFAD2* genes was investigated by sequence alignment and phylogenetic analysis.

### Transcriptome analyses

We did two transcriptome analyses. One aimed to investigate the expression profiles of individual *GhFAD2* in various tissues and the other compared the difference in anther transcriptomes between the *GhFAD2–3* silenced transgenics and wild-type. For the first experiment, total RNA was isolated from root, leaf, stem, anther, stigma, ovary, developing and mature seed (at 5, 20, 40 and 60 days post anthesis) and fiber (at 12 and 24 days post anthesis). Each sample had three biological replicates. For each sample, a total of 3 μg RNA was used in preparing the RNA-seq library. Barcoded multiplexed RNA-seq libraries were created using the NEBNext® Ultra™ RNA Library Prep Kit for Illumina® (NEB, USA) according to the manufacturer’s protocol. Clean paired-end reads were aligned to the TM-1 reference genome [35], and the number of reads aligned to each gene was measured using HTSeq v0.6.1. The expression levels of individual genes were quantified using FPKM (fragments per kilobase of transcript per million mapped reads).

For the anther transcriptome experiment, total RNA was isolated from anthers of wild-type (control) and transgenic plants with silenced *GhFAD2–3* (*fad2–3*). Anthers from two developmental stages, meiotic and tetrad, were investigated. Three samples were collected from each stage for both wild-type and *fad2–3*. A total amount of 3 μg RNA from each sample was used in generating index-coded RNA-seq libraries using the Illumina TruSeq RNA Library Prep Kit. The clustering of the index-coded libraries was performed using the TruSeq PE Cluster Kit v3-cBot-HS (Illumina) on a cBot Cluster Generation System following the manufacturer’s manual. The libraries were then sequenced using the Illumina HiSeq 2500 platform. Read mapping and FPKM calculations were performed as described previously. The model based on negative binomial distribution was used to determine differentially expressed genes (DEG) with an adjusted *p*-value < 0.05 [52]. The KOBAS software was used to test statistically the enrichment of DEGs in KEGG pathways.

### Plant material and generation of RNAi plants

The cotton variety ‘Xinluzao 33’ provided by the Cotton Research Institute of Shihezi University was used in this study. A 517-bp fragment (Additional file 1: Figure S1) was amplified by PCR using PrimeSTAR™ HS DNA polymerase. The primers used were 5′-*CACC*CGCTCACTTATCCGTTCA-3′ (CACC was added at the 5′ end for directional cloning of the amplified fragment into the RNAi construct) and 5′-CGTTGTAGATAGGACCGTAT-3′. The PCR cycles were performed as follows: 95 °C for 5 min, followed by 29 cycles at 94 °C for 45 s, 55 °C for 45 s, 72 °C for 45 s, and a final extension at 72 °C for 10 min. The amplified DNA fragment was subcloned into pENTR/D-TOPO (Invitrogen) to generate the entry vector pENTR/D-*GhFAD2* that was confirmed by sequencing. The LR recombination reaction between pENTR/D-*GhFAD2* and the gateway vector pANDA35HK was then used to create pANDA35HK-*dsGhFAD2* using the Gateway™ LR Clonase™ plus enzyme mix (Invitrogen). The native promoter of *GhFAD2–3D* isolated from cotton genomic DNA (Additional file 1: Figure S2a) was cloned into the *Hin* dIII-*Bam* HI site of pBI121 to generate pBIAP. Finally, the fragment with the 517-bp fragment inserted forwardly and reversely at two sides of the intron was excised from pANDA35HK-*dsGhFAD2* and used to replace the *GUS* gene in pBIAP to create pBIAP-dsGhFAD2 (Additional file 1: Figure S2b), which was then electroporated into the *Agrobacterium tumefaciens* strain LBA4404. Cotton transformation was carried out using hypocotyl explants from *G. hirsutum* cultivar Xinluzao 33 as described by Jin et al. [53].

A fragment (1,113 bp) containing part of the 517-bp *GhFAD2–3* segment and part of the *gus* linker was amplified by PCR to identify positive transgenic plants. Genomic DNA was isolated from cotton leaf tissues and used in PCR amplification using the primers, 5′-CTGTACAGCGAAGAGGCAGTC-3′ and 5′-CGTTGTAGATAGGACCGTAT-3′. The pair of primers 5′-GAGTCTGGTAATTGGAATGAG-3′ and 5′-TTCGCAGTTGTTCGTCTT-3′ was used to amplify the 18S rDNA gene as a control. Cultivar ‘Xinluzao 33’ and transgenic cotton plants were grown in soil in a growth room with a 16 L:8D photoperiod and 50% relative humidity.

### Phenotypic analysis and pollen staining

Plants or flowers were photographed with a digital camera (Canon, Japan) or a SteREO Discovery microscope (Carl Zeiss). In all experiments, phenotypes of the RNAi plants (*fad2–3*) were analyzed together with wild-type. For testing pollen viability, pollen grains were stained with 1% I_2_-KI solution and photographed using a SteREO Discovery microscope (Carl Zeiss). For the control plants, sufficient mature pollens were obtained by placing 3–4 open flowers in a microfuge tube. For the *fad2–3* plants, mature anthers were dissected from flowers and gently squashed in staining solution using dissecting needles. Acetolysis treatment was carried out according to Aarts et al. [19], and pollen grains were treated with a mixture of sulfuric acid and acetic anhydride at 100 °C. The pollen pellet was then transferred to a microscope slide and viewed with a SteREO Discovery microscope.

### Scanning and transmission electron microscopy

For scanning electron microscopy, anthers at different developmental stages were fixed overnight in 2.5% glutaraldehyde, and then washed and postfixed in 1% osmium tetraoxide in 0.1 M sodium phosphate buffer (PBS, pH 7.2). Samples were then dehydrated in a graded ethanol concentration (30, 50, 70, 95 and 100%) and dried with liquid CO_2_. Before examination with the scanning electron microscope, dried anthers were sputtered with gold palladium for 300 s at 25 mA. Samples were finally visualized using a Hitachi S4500 microscope.

Transmission electron microscopy was performed using a Hitachi H7600 transmission electron microscope. Anthers were fixed in 2.5% glutaraldehyde (stored overnight at 4 °C), washed three times (5 min for each) with 0.1 M PBS, postfixed in 1% OsO_4_ for 2 h, and washed with PBS (three times, 5 min for each). Samples were then dehydrated as described above, treated with propylene oxide, and embedded in Spurr’s resin. Thin sections (70 nm) were taken using the Leica UC6 cryo ultramicrotome. Sliced sections were placed on 100-mesh copper grids and sequentially stained with uranyl acetate (30 min) and lead citrate (Sato’s Lead; 15 min).

### Anther collection and fatty acid analysis

To analyze the composition of fatty acids in developing anthers, different sizes of flower buds (without bracts) with a diameter < 9 mm were collected from wild-type (fertile) and *fad2–3* (sterile) and used in isolation of anthers with pollen grains at the following five developmental stages: sporogenous cells, microsporocyte, meiosis, tetrad and pollen maturation. Identification of these developmental stages was done using optical microscopy.

The whole anther (including both anther wall and pollen grains) of the five stages mentioned above was used in fatty acid analysis. The fatty acid methyl esters were prepared by alkaline transmethylation. Briefly, 0.5 g freeze-dried anthers were transferred into glass tubes, and used in oil extraction by using a Soxtherm apparatus (Gerhadt). Then, 5 ml 0.4 M KOH-methanol and 5 ml hexane were added and mixed. The solution was transferred into vials and shaken for 30 min at 40 °C. After adding ~ 1 g of anhydrous sodium sulfate to remove water, the upper hexane layer was used in GC-MS analysis. The quantitative standard curve was established by mixing 37 fatty acid methyl esters (Sigma) determined by GC-MS, and the external standard method was used for quantitative determination. The analyses were performed using GCMS-QP2020 at an electron ionization of 70 eV with an HP-88 capillary column (100 m × 0.2 mm) and film thickness of 0.2 μm. The column program used was: the injection temperature 250 °C, oven temperature kept at 40 °C for 2 min, then increased to 240 °C at a rate of 4 °C/min, and kept constant at 240 °C for 15 min. Operating conditions: helium carrier gas 2 mL/min, split ratio 10:1.

### Analysis of anther cuticular waxes and cutin-like polyester

The wax of anther at mature pollen stage was analyzed using a published protocol [29] with some modifications. Briefly, 100 mg of freeze-dried anthers was submersed in 10 ml of chloroform containing 100 μg of tetracosane (Fluka; serving as an internal standard) for 1 min. The solution was transferred to a new vial, and then the solvent was evaporated under a nitrogen gas stream. The remaining compounds were incubated with 200 μl bis-N,N-(trimethylsilyl)-trifluoroacetamide (Sigma-Aldrich) in 200 μl pyridine for 60 min at 70 °C before GC-MS analysis. The constituent analyses were performed using GCMS-QP2020 with a DB-1 column of 30 m × 0.32 mm and film thickness of 0.1 μm. GC-MS analyses were performed as described by Jung et al. [29]. Each compound was quantified against the internal standard by automatic integration of the peak areas.

The protocol for lipid polyester analysis was performed according to Li-Beisson et al. [54]. First, 100 mg of freeze-dried anthers was delipidated. After that, depolymerization was performed by acid catalysis as described by Li-Beisson et al. [50]. The resulting cutin monomer fraction was derivatized with BFTSA/pyridine (1:1) for 60 min at 70 °C, and then samples were analyzed using GCMS-QP2020 with a DB-1 column of 30 m × 0.32 mm and film thickness of 0.1 μm. The GC-MS was conducted according to Li-Beisson et al. [50] with helium carrier gas at 2 ml/min. Each compound was quantified on the basis of their total ion current as described by Li-Beisson et al. [54].

### Quantitative real-time PCR (qRT-PCR) analysis

Total RNA was extracted from anthers at different developmental stages using RNAiso Plus (Takara), and then the RNA was reverse transcribed to obtain first-strand cDNA using a PrimeScript™ 1st Strand cDNA Synthesis Kit (Takara, China). The transcript levels of genes were analyzed by qRT-PCR using the LightCycler® 480 II (Roche, Germany). Each reaction was performed in 10 μl volumes using SYBR Green Master Mix (Takara, China) under the following PCR conditions: 94 °C for 3 min followed by 40 cycles of 94 °C for 15 s, 56 °C for 15 s, and 72 °C for 15 s. All gene specific primers for qRT-PCR were designed using the Primer 6.0 program (Additional file 1: Table S2). The cotton poly-ubiquitin gene (*GhUBQ14*, accession number in GenBank: DW505546) was used as an internal control. The primers for *GhUBQ14* were 5′-CAACGCTCCATCTTGTCCTT-3′ and 5′-TGATCGT CTTTCCCGTAAGC-3′. All qRT–PCR reactions were performed in triplicate. The relative expression levels of target genes were calculated with the 2^–ΔΔCt^ method [55].

## Additional files

Additional file 1: **Figure S1.** The coding sequences and phylogenetic analysis of *GhFAD2.* **A.** The coding sequences of *GhFAD2* genes. The fragment in *GhFAD2–3D* and *GhFAD2–3A* targeted for RNAi is highlighted in red color. The underlined sequence in *GhFAD2–4A* was filled up in this study, which is a gap in the TM-1 genome (Zhang et al. 2015). **B.** Putative members of the *GhFAD2* family in the TM-1 genome identified based on blastp search using the protein sequence of the published GhFAD2–1 (X97016). Gh_D13G2237 contains three indels (50, 14 and 21 aa, respectively) compared to other proteins, and its 3rd deletion contains the 3rd conserved histidine-cluster observed in all FAD2 protein, this gene was thus considered as a non-functional FAD2 and not analysed further in this study. The three conserved histidine-clusters are highlighted in red. The annotated Gh_A01G2091 was incomplete due to gap in the genomic sequence, and the missing sequence was filled up by sequence cloning in this study. **C.** Phylogenetic analysis of the cotton FAD2 family. The tree was generated based on protein sequences of cotton FAD2 using the Maximum likelihood module of the MEGA6 software. **Figure S2** Generation and molecular analysis of transgenic plants. **A.** Schematic representation of the pBIAP-ds*GhFAD2* expression cassettes used for cotton transformation. Promoter, Anther-specific promoter; NPT II, *neomycin phosphotransferase II* gene; *GhFAD2*, The partial fragment *GhFAD2–3D* coding sequence; RB, right border; LB, left border; **B.** Generation of transgenic plants. a, induction of embryonic calli; b, calli produced from explants; c, embryoid produced from calli; d, regeneration of kanamycin resistant plantlets. **C.** Detection of the *GhFAD2-gus linker* fusion fragment in non-transformed control and transgenic plants by PCR. A 1113-bp fusion fragment was amplified and 18 s rDNA was served as a control. The primers used in amplification were 5′-CTGTACAGCGAAGAGGCAGTC-3′ and 5′-CGTTGTAGATAGGACCGTAT-3′ for *GhFAD2-gus linker* fusion fragment, and 5′-GAGTCTGGTAATTGGAATGAG-3′ and 5′-TTCGCAGTTGTTCGTCTT-3′ for 18S rDNA. Lane M, DNA marker III; Lane 1–4; 18 s rDNA sequence was amplified from transformants line; Lane 5, 18 s rDNA sequence was amplified from untransformed control; Lane 6–9, The fusion fragment was amplified from transformants line; Lane 10, The fusion fragment was amplified from untransformed control. **Table S1.** Representative differentially expressed genes involved in anther lipid metabolism. **Table S2.** Primers of selected genes used for qRT-PCR analysis. (DOCX 1056 kb)

Additional file 2: Raw data. This file contains raw data with individual data points or replicates for Figs. 1, 5, 6, and 7. (XLS 126 kb)

## Data Availability

All Gene ID and annotation files could be obtained from CottonGen (https://www.cottongen.org). Raw data for Figs. 1, 5, 6, and 7 can be found in ‘Additional file 2: Raw data’. All other data generated or analyzed during this study are included in this manuscript.

## Abbreviations

C14:0: Myristic acid
C16:0 DCA: Hexadecane-1,16-dioic acid
C16:0: Palmitic acid
C18:0: Stearic acid
C18:1 DCA: α, ω-octadecenoic acid
C18:1: 9-octadecenoic acid
C18:2 DCA: α, ω-octadecadiendioic acid
C18:2: 9,12-octadecadienoic acid
C18:3: 9,12,15-octadecatrienoic acid
C20:0: Arachidic acid
DPA: Days post anthesis
DW: Dry weight
ER: Endoplasmic reticulum
FAD2: Fatty acid desaturase 2
FAD3: Fatty acid desaturase 3
FAEH: Fatty acid epoxide hydrolase
FAR: Fatty acyl-coenzyme A Reductase
FAS: Fatty acid synthase
FAs: Fatty acids
HTH: ω-hydroxyacid dehydrogenase
NPT II: Neomycin phosphotransferase II gene
OADH: ω-oxo-acid dehydrogenase
P450: Cytochrome P450 monooxygenase
PUFAs: Polyunsaturated fatty acids
PXG: Peroxygenase
SAD: Stearoyl-ACP desaturase
triOH C18:1 FA: 9,10,18-trihydroxy octadecenoic acid
