# Supplementary material for: GhFAD2–3 is required for anther development in Gossypium hirsutum
Source: BMC Plant Biol. 2019 Sep 10;19:393. doi: 10.1186/s12870-019-2010-9 (PMC6734329; doi:10.1186/s12870-019-2010-9)
Supplement: Supplementary file 1 — Figure S1. The coding sequences and phylogenetic analysis of GhFAD2. A. The coding sequences of GhFAD2 genes. The fragment in GhFAD2–3D and GhFAD2–3A targeted for RNAi is highlighted in red color. The underlined sequence in GhFAD2–4A was filled up in this study, which is a gap in the TM-1 genome (Zhang et al. 2015). B. Putative members of the GhFAD2 family in the TM-1 genome identified based on blastp search using the protein sequence of the published GhFAD2–1 (X97016). Gh_D13G2237 contains three indels (50, 14 and 21 aa, respectively) compared to other proteins, and its 3rd deletion contains the 3rd conserved histidine-cluster observed in all FAD2 protein, this gene was thus considered as a non-functional FAD2 and not analysed further in this study. The three conserved histidine-clusters are highlighted in red. The annotated Gh_A01G2091 was incomplete due to gap in the genomic sequence, and the missing sequence was filled up by sequence cloning in this study. C. Phylogenetic analysis of the cotton FAD2 family. The tree was generated based on protein sequences of cotton FAD2 using the Maximum likelihood module of the MEGA6 software. Figure S2 Generation and molecular analysis of transgenic plants. A. Schematic representation of the pBIAP-dsGhFAD2 expression cassettes used for cotton transformation. Promoter, Anther-specific promoter; NPT II, neomycin phosphotransferase II gene; GhFAD2, The partial fragment GhFAD2–3D coding sequence; RB, right border; LB, left border; B. Generation of transgenic plants. a, induction of embryonic calli; b, calli produced from explants; c, embryoid produced from calli; d, regeneration of kanamycin resistant plantlets. C. Detection of the GhFAD2-gus linker fusion fragment in non-transformed control and transgenic plants by PCR. A 1113-bp fusion fragment was amplified and 18 s rDNA was served as a control. The primers used in amplification were 5′-CTGTACAGCGAAGAGGCAGTC-3′ and 5′-CGTTGTAGATAGGACCGTAT-3′ for GhFAD2-gus linker fusion fragme [file 12870_2019_2010_MOESM1_ESM.docx]

**(A)**

**>***GhFAD2-1A, Gh_A13G1850*

ATGGGTGCCGGTGGTAGGATGCTAGTTGACGGTAAAAAGGAGGAAAAACGAGGCTCGGTCAATCGAGTTCCGATCGAGAAGCCTCCGTTTACGCTCGGTCAGATCAAGCAAGCCATTCCGCCCCACTGTTTTCGCCGCTCCCTCCTTCGATCCTTCTCCTACGTGGTCCATGACCTATGCTTAGCCTCTCTCTTTTACTACATTGCAACATCATATTTTCACTTTCTCCCACAACCCTTTTCCTACATTGCTTGGCCTGTCTATTGGGTTCTCCAAGGTTGCATCCTCACCGGTGTTTGGGTCATCGCACACGAGTGCGGTCACCACGCTTTCAGTGACTACCAATGGGTTGACGACACCGTCGGGTTGATCCTTCATTCCGCCCTTTTAGTCCCGTACTTCTCGTGGAAAATCAGTCACCGCCGTCACCACTCGAACACCGGTTCCATGGAGCGTGACGAAGTATTCGTGCCCAAACCCAAGTCTAAATTATCATGCTTTGCGAAATACTTAAACAATCCACCCGGTCGAGTTCTATCTCTTGTAGTCACATTGACTCTTGGTTGGCCTATGTACTTAGCCTTCAACGTTTCGGGTCGATACTATGATCGATTAGCTTCCCACTATAACCCTTATGGCCCCATTTACTCCGATCGCGAGAGGCTACAAGTTTACATCTCCGATACTGGTATATTTGCGGTAATTTATGTACTTTATAAGATTGCTGCAACAAAAGGGCTGGCTTGGCTTTTATGCACTTATGGGGTGCCTCTACTTATTGTGAATGCCTTCCTTGTGTTGATCACCTACTTGCAACATACTCACTCGGCATTGCCGCATTATGACTCGTCCGAATGGGATTGGTTGCGAGGAGCATTGTCGACGATGGATCGAGATTTCGGGGTGTTGAACAAAGTGTTCCATAACATCACCGATACGCATGTTGCTCATCACCTCTTCTCAACGATGCCACATTATCATGCAATGGAGGCCACTAAAGCAATCAAACCAATACTCGGCAAGTATTATCCTTTCGACGGGACACCGATTTACAAGGCAATGTGGAGGGAGGCAAAAGAGTGCCTTTACGTTGAGCCTGACGTTGGTGGTGGTGGTGGTGGTAGCAAAGGTGTTTTTTGGTATCGTAACAAGTTCTAA

*>GhFAD2-1D, Gh_D13G2238*

ATGGGTGCCGGTGGTAGGATGCCAATTGACGGTATAAAGGAGGAAAATCGAGGCTCGGTCAATCGAGTTCCGATCGAGAAGCCTCCGTTTACGCTCGGTCAGATCAAGCAAGCCATTCCGCCCCACTGTTTTCGCCGCTCCCTCCTTCGATCCTTCTCCTACGTGGTCCATGACCTATGCTTAGCCTCTCTCTTTTACTACATTGCAACATCATATTTTCACTTTCTCCCACAACCCTTTTCCTACATTGCTTGGCCTGTCTATTGGGTTCTCCAAGGTTGCATCCTCACCGGTGTTTGGGTCATCGCACACGAATGCGGTCACCACGCTTTCAGTGACTACCAATGGGTTGACGACACCGTCGGGTTGATCCTTCACTCCGCCCTTTTAGTCCCGTACTTCTCGTGGAAAATCAGTCACCGCCGTCACCACTCGAACACCGGTTCCATGGAGCGTGACGAAGTATTCGTGCCCAAACCCAAGTCTAAATTATCATGCTTTGCGAAATACTTCAACAATCCACCCGGTCGAGTTCTCTCTCTTGTAGTCACATTGACTCTTGGTTGGCCTATGTACTTAGCCTTCAACGTTTCGGGTCGATACTATGATCGATTAGCTTCCCACTATAACCCTTACGGCCCCATTTACTCCGAACGCGAGAGGCTACAAGTTTACATCTCCGATGCTGGTATAGTTGCGGTAATTTATGTACTTTATAAGATTGCTGCAACAAAAGGGCTGGCTTGGCTTTTATGCACTTATGGGGTACCTCTACTTATTGTGAATGCCTTCCTTGTGTTGATCACCTACTTGCAACATACTCACTCGGCATTGCCGCATTACGACTCGTCTGAATGGGATTGGTTTCGAGGAGCATTGTCGACGATTGATCGAGATTACGGGGTGTTGAACAAAGTGTTCCATAACATCACCGATACGCATGTGGCTCATCACCTCTTCTCAACGATGCCACATTATCATGCAATGGAGGCCACTAAAGCAATCAAACCGATACTCGGCAAGTATTATCCTTTCGACGGGACACCGATTTATAAGGCAATGTGGAGGGAGGCAAAAGAGTGCCTTTACGTCGAGGCTGACGTTGGTGGTGGTGGTAGCAAAGGTGTTTTTTGGTATCGTAACAAGTTCTAA

*>GhFAD2-2A, Gh_A01G2094*

ATGTCGGTTCCACCGAGTCCAAAAAAATCCGAATTCAACTCACTGAAGCGAGTTCCATACTCAAAGCCGCCCTTCACTCTGAGTGAAATCAAGAAAGCCATCCCACCACACTGTTTCCAGCGCTCCGTTTTACGCTCATTCTCATATCTCCTTTACGACTTTATATTGGCCTCTCTTTTCTATTATGTGGCCACCAATTACTTCCATAACCTTCCTCAGCCTCTCTCCTACGTGGCTTGGCCTCTTTATTGGGCCATGCAAGGTTGGATTTTGACCGGCGTTTGGGTCATAGCCCATGAATGTGGCCACCATGCCTTCAGTGATTATCAATGGCTTGACGACACCGTTGGCCTTATCCTCCACTCTTCTCTCTTAGTTCCATATTTCTCTTGGAAATATAGCCACCGTCGTCACCATTCCAATACCGGTTCCCTCGAAAGGGATGAAGTGTTCGTTCCCAAGAAAAAATCTGGTTTAAGATGGTGGGCCAAACACTTCAACAATCCACCGGGTCGGTTTCTGTCAATCACCATTCAACTTACCCTTGGTTGGCCGCTTTACTTAGCTTTCAACGTTGCCGGTCGGCCTTACGACAGGTTCGCTTGCCACTATGACCCTTACGGCCCCATATTTTCCGACCGGGAACGACTCCAAATCTATATCTCTGACGCCGGCGTCCTCGCTGTCGCCTATGCGCTCTACCGTCTCGTGTTGGCCAAAGGGGTAGGTTGGGTTATTAGCGTTTATGGGGTGCCATTATTGGTGGTTAACGCCTTCTTAGTAATGATCACGTATTTGCAACACACTCACCCATCTTTGCCGCACTATGATTCCTCGGAGTGGGACTGGATGAGAGGAGCTTTATCAACTGTGGACAGAGATTATGGGATTTTAAACAAGGTTTTCCATAACATAACCGACACTCATGTGGCTCATCATTTGTTTTCTACAATGCCTCACTATCATGCAATGGCGGCCACCAAGGCGATAAAGCCAATATTGGGGGAATACTATCAGTTCGATGGGATGCCTGTCTATAAGGCGATATGGAGGGAGGCGAAGGAGTGTCTCTACGTTGAACCAGATGAGGGCGACAAGGATAAAGGTGTGTTTTGGTTTAGAAACAAGCTTTAA

**>***GhFAD2-2D, Gh_D01G1227*

ATGTCGGTTCCAACGAGTCCAAAAAAACCCGAATTCAACTCACTGAAGCGAGTTCCATACTCAAAGCCACCCTTCACTCTGAGTGAAATCAAGAAAGCCATCCCACCACACTGTTTCCAGCGCTCCGTTTTACGCTCATTCTCATATCTCCTTTACGACTTTATATTGGCCTCTCTTTTCTACCATGTGGCCACCAATTACTTCCCTAACCTTCCTCAGGCTCTCTCCAACGTGGCTTGGCCTCTTTATTGGGCCATGCAAGGTTGCATTTTGACCGGCGTTTGGGTCATAGCCCATGAATGTGGCCACCATGCTTTCAGTGATTATCAATGGCTTGACGACACCGTGGGCCTTATCCTCCACTCTTCTCTCTTAGTTCCATATTTCTCTTGGAAATATAGCCACCGGCGTCACCATTCTAACACCGGTTCCCTCGAAAGGGATGAAGTGTTCGTTCCCAAGAAAAAATCTGGTTTAAGATGGTGGGCCAAACACTTCAACAATCCACCGGGTCGGTTTCTGTCAATCACCATTCAACTTACCCTTGGTTGGCCGCTTTACTTAGCTTTCAACGTTGCCGGCCGGCCTTACGACAGGTTCGCTTGCCACTATGACCCTTACGGCCCCATATTTTCCGACCGGGAACGACTCCAAATCTATATCTCTGACGCCGGCGTCCTCGCTGTCGCCTATGCGCTCTACCGTCTCGTGTTGGCCAAAGGGGTAGGTTGGGTTATTAGCGTTTATGGGGTGCCATTATTGGTGGTTAACGCCTTCTTAGTAATGATCACGTATTTGCAACACACTCACCCATCTTTGCCGCACTATGATTCCTCGGAGTGGGACTGGATGAGAGGAGCTTTATCAACTGTGGACAGAGATTATGGGATTTTAAACAAGGTTTTCCATAACATAACCGACACTCATGTGGCTCATCATTTGTTTTCGACAATGCCTCACTATCATGCCATGGTGGCCACCAAGGCGATAAAGCCAATATTGGGGGAATACTATCAGTTCGATGGGATGCCTGTCTATAAGGCGATATGGAGGGAGGCGAAGGAGTGTCTCTACGTTGAACCAGATGAGGGCGACAAGGATAAAGGTGTGTTTTGGTTTAGAAACAAGCTTTAA

**>***GhFAD2-3A, Gh_A11G2814*

ATGGGTGCAGGTGGCAGAATGTCGGTTCCTCCAAGTCAAAGGAAACAAGAATCGGGCTCAATGAAAAGAGTCCCTATATCTAAACCACCATTTACTCTCAGTGAAATAAAAAAAGCCATCCCACCACACTGTTTCCAACGCTCACTTATCCGTTCATTTTCCTATCTCGTTTACGACTTCATTTTAGTCTCTATCTTTTACTACGTAGCCACCACTTACTTCCACAACCTCCCTCAGCCACTATCTTTCGTCGCCTGGCCAATTTATTGGACTCTTCAAGGTTCAGTCCTCACTGGCGTTTGGGTTATCGCCCATGAATGCGGTCACCATGCTTTTAGCGATTACCAATGGATTGATGACACTGTCGGTCTCATCCTCCATTCATCCCTTCTTGTCCCGTACTTTTCGTGGAAATATAGTCACCGACGTCACCATTCCAACACTGGTTCCCTTGAACGCGACGAAGTATTTGTTCCGAAGAAACGGAGCAGCATTAGATGGTGGGCTAAATACCTCAACAATCCACCAGGTCGTTTCGTCACAGTCACCATTCAGCTCACTCTCGGATGGCCTCTTTACTTAGCATTCAATGTAGCAGGTAGACCTTACGAAGGACTCGCTTGTCACTACAACCCATACGGTCCTATCTACAACGACCGTGAACGACTTCAAATCTACATATCCGACGTCGGTGTCCTTGCTGTCACCTATGGGCTGTACCGTCTCGTGTTAGCCAAAGGTCTAGCTTGGGTCATTTGCGTTTACGGTGTCCCATTGCTCATCGTTAATGCATTCCTCGTCATGATCACATACTTGCAACACACTCACCCCGCATTACCACACTACGACTCATCCGAATGGGACTGGTTACGTGGAGCCCTCGCGACGGTCGACCGAGATTATGGGATATTAAACAAGGTTTTCCATAACATAACTGATACTCATGTCGCTCATCATTTGTTTTCGACGATGCCGCATTACCACGCAATGGAAGCAACTAAGGCAATAAAACCAATATTGGGAGAGTATTATTCATTTGATGGTACACCAGTTTATAAAGCGATATTTAGAGAGGCAAAGGAGTGTATTTACGTTGAACCAGACGAAGGTGAGCAGAGCAGCAAAGGTGTATTTTGGTTTAGAAATAAGATCTAA

**>***GhFAD2-3D, Gh_D11G3169*

ATGGGTGCAGGTGGCAGAATGTCGGTTCCTCCAAGTCAAAGGAAACAAGAATCGGGCTCAATGAAAAGAGTCCCTATATCTAAACCACCATTTACTCTCAGTGAAATAAAAAAAGCCATCCCACCACACTGTTTCCAACGCTCACTTATCCGTTCATTTTCCTATCTCGTTTACGACTTCATTTTAGTCTCTATCTTTTACTACGTAGCCACCACTTACTTCCGCAACCTCCCTCAGCCACTATCTTTCGTCGCCTGGCCAATTTATTGGGCTCTTCAAGGTTCAGTCCTCACTGGCGTTTGGGTTATCGCCCATGAATGCGGTCACCACGCTTTTAGCGATTACCAATGGATCGATGACACTGTCGGTCTCATCCTCCATTCATCCCTTCTCGTCCCGTACTTTTCGTGGAAATATAGTCACCGTCGTCACCATTCCAACACTGGTTCCCTTGAACGCGACGAAGTATTTGTTCCGAAGAAACGGAGCAGCATTAGATGGTGGGCTAAATACCTCAACAATCCACCAGGTCGTTTCGTCACAATCACCATTCAGCTCACTCTCGGATGGCCTCTTTACTTAGCATTCAATGTAGCAGGTAGACCTTACGAAGGATTCGCTTGTCACTACAACCCATACGGTCCTATCTACAACGACCGTGAACGACTTCAAATCTACATTTCCGACGTCGGTGTCCTTGCTGTCACCTATGGGCTGTACCGTCTCGTGTTAGCCAAAGGTCTAGCTTGGGTCATTTGTGTTTACGGTGTCCCATTGCTCATCGTTAATGCATTCCTCGTCATGATCACATACTTGCAACACACTCACCCTGCATTACCACACTACGACTCATCCGAATGGGATTGGTTACGTGGAGCCCTCGCGACGGTCGACCGAGATTATGGGATATTAAACAAGGTTTTCCATAACATAACTGATACTCATATCGCTCATCATTTGTTTTCGACAATGCCGCATTACCACGCAATGGAAGCAACAAAGGCAATAAAGCCAATATTGGGCGAGTATTATTCATTTGATGGTACACCAGTTTATAAAGCGATATTTAGAGAGGCAAAGGAGTGTATTTACGTTGAACCAGACGAAGGTGAGCAGAGCAGCAAAGGTGTATTTTGGTTTAGAAATAAGATCTAA

**>***GhFAD2-4A, Gh_A01G2091*

ATGGGTGCTGGAGGTAGAACGTCAGTTCCACCGAGTCCCAAAAAACCCGAATTCTACCCACTGAAGCGAGTTCCATGCTCAAAGCCACCCTTCACTCTGAGTCAAATAAAAAAAGCCATCCCACCTCACTGTTTCAAGCGCTCCGTTTTACGCTCATTCTCTTATCTTATTTACGACCTTGTATTGGCCTCTCTTTTCTACTATGTGGCCACCAATTACTTCCCTAGCCTTCCTCAACCTCTCCCGTATGTGGCTTGGCCTCTTTACTGGGCCATGCAAGGTTGTATTTTCACCGCCTTTTGGGCGCTTGCCCATGAATGTGGTCATCAAGCTTTCAGTGATTATCAATGGCTTGACGACACTATTGGCTTTATCCTCCACACTTTTCTCTTAACTCCATATTTCTCGTTGAAATATAGCCATCGCCGTCACCATTCCAACACTGGTTCCCTCGAAAGGGACGAAGTCTTCGTTCCAAAGAAAAAATCCGCTTTAAAATGGTGGGCTAAACACTTCAATAATCCACTAGGCCGGTTTTTGGAAATCTCCATTCAACTTACCCTCGGCTGGCCGCTTTACTTACTGTTCAACATCACTGGTCCACCTTACGATAGGTTAGCTTGCCACTATGACCCTTATGGCCCCATCTTTTCCGACCGCCAACGGCTCCAAATATACGTCTCCGACGCCGGCGTCCTCGCCGTCACCTATGCTCTTTACCGTCTCGTGTTGGCCAAAGGGGTAGGTTGGGTTATTAGCGTTTACGGGGTGCCATTATTGGTGGTTAATGCCTTCTTAGTAATGATCACGTATTTGCAACACACTCACCCATCTTTGCCGCACTATGAGTCTTCGGAGTGGGACTGGTTGAGGGGAGCTTTATCGACCGTGGACAGAGATTATGGGATTTTAAACAAGGTTTTCCATGACATAACCCGCATACACGTGGCTCACCATTTGTTTCCGACAATACCTCACTATCATGCAATGGAGGCTACCAAGGTGATAAAGCCGGTTTTGGGGGAATACTACCAGTTCGATGGGACACCCGTTTATAAGGCGATATGGAGGGAGTTGAAGGAGTGTGTTTACGTTGAACCAGATGAGGGTGATAATGATAAAGGTGTTTTATGGTTTAGAAACAAGCTTTAA

**>***GhFAD2-4D, Gh_D01G1226*

ATGGGTGCTGGAGGTAGAACATCGGTTCCACCGAGTCCCAAAAAACCCGAATTCAACCCACTGAAGCGAGTTCCATGCTCAAAGCCACCCTTCACTCTGTGTCAAATAAAAAAAGCCATCCCACCTCACTGTTTCAAGCGCTTCGTTTTACGCTCATTCTCTTATCTTATTTACGACCTTGTATTGGCCTCTCTTTTCTACTATGTGGCCACCAATTACTTCCCTAGCCTTCCTCAAGCTCTCCCGTATGTGGCTTGGCCTATTTATTGGGCCATGCAAGGTTGTATTTTCACCGCCTTTTGGGCGCTTGCCCATGAATGTGGTCATCAAGCTTTCAGTGATTATCAATGGCTTGACGACACTATTGGCTTTATCCTCCACACTTTTCTATTAACTCCATATTTCTCGTTGAAATATAGCCATCGCCGTCACCATTCCAACACTGGTTCCCTCGAAAGAGACGAAGTCTTCGTTCCAAAGAAAAAATCCGCTTTAAAATGGTGGGCTAAACACTTCAATAATCCACTAGGCCGGTTTTTGGAAATCTCCATTCAACTTACCCTCGGCTGGCCGCTTTACTTACTGTTCAACATCACTGGTCCACCTTACGATAGGTTAGCTTGCCACTATGACCCTTATGGCCCCATCTTTTCCGACCGCCAACGGCTCCAAATATACGTCTCCGACGCCGGCGTCCTCGCTGTCACCTATGCTCTTTACCTTCTCGTGTTGGCCAAAGGGGTAGGTTGGGTTATTAGCGTTTACGGGGTGCCATTATTGGTGGTTAATGCCTTCTTAGTAATGATCACGTATTTGCAACACACTCACCCATCTTTGCCGCACTATGAGTCTTCGGAGTGGGACTGGTTGAGGGGAGCTTTATCGACCGTGGACAGAGATTATGGGATTTTAAACAAGGTTTTCCATGACATAACCCGCATACACGTGGCTCACCATTTGTTTCCGACAATACCTCACTATCATGCAATGGAGGCTACCAAGGTGATAAAGCCGGTTTTGGGGGAATACTACCAGTTCGATGGGACACCCGTTTATAAGGCGATATGGAGGGAGTTGAAGGAGTGTGTTTACGTTGAACCAGATGCGGGTGATAATGATAAAGGTGTTTTATGGTTTAGAAACAAGCTTTAA

**(B)**

Gh_D13G2237 ------MVVHGKKNKENRGWVNRVSIKKPPFTLSQIKQDILPHC---------------- 38

Gh_A13G1850 MGAGGRMLVDG-KKEEKRGSVNRVPIEKPPFTLGQIKQAIPPHCFRRSLLRSFSYVVHDL 59

Gh_D13G2238 MGAGGRMPIDG-IKEENRGSVNRVPIEKPPFTLGQIKQAIPPHCFRRSLLRSFSYVVHDL 59

Gh_A01G2091 MGAGGRTSVPPSPKKPEFYPLKRVPCSKPPFTLSQIKKAIPPHCFKRSVLRSFSYLIYDL 60

Gh_D01G1226 MGAGGRTSVPPSPKKPEFNPLKRVPCSKPPFTLCQIKKAIPPHCFKRFVLRSFSYLIYDL 60

Gh_A11G2814 MGAGGRMSVPPSQRKQESGSMKRVPISKPPFTLSEIKKAIPPHCFQRSLIRSFSYLVYDF 60

Gh_D11G3169 MGAGGRMSVPPSQRKQESGSMKRVPISKPPFTLSEIKKAIPPHCFQRSLIRSFSYLVYDF 60

Gh_A01G2094 ------MSVPPSPKKSEFNSLKRVPYSKPPFTLSEIKKAIPPHCFQRSVLRSFSYLLYDF 54

Gh_D01G1227 ------MSVPTSPKKPEFNSLKRVPYSKPPFTLSEIKKAIPPHCFQRSVLRSFSYLLYDF 54

: .: : ::** .****** :**: * ***

Gh_D13G2237 ----------------------------------CILTGVWVIAHECGHHAFSDYQWVDN 64

Gh_A13G1850 CLASLFYYIATSYFHFLPQPFSYIAWPVYWVLQGCILTGVWVIAHECGHHAFSDYQWVDD 119

Gh_D13G2238 CLASLFYYIATSYFHFLPQPFSYIAWPVYWVLQGCILTGVWVIAHECGHHAFSDYQWVDD 119

Gh_A01G2091 VLASLFYYVATNYFPSLPQPLPYVAWPLYWAMQGCIFTAFWALAHECGHQAFSDYQWLDD 120

Gh_D01G1226 VLASLFYYVATNYFPSLPQALPYVAWPIYWAMQGCIFTAFWALAHECGHQAFSDYQWLDD 120

Gh_A11G2814 ILVSIFYYVATTYFHNLPQPLSFVAWPIYWTLQGSVLTGVWVIAHECGHHAFSDYQWIDD 120

Gh_D11G3169 ILVSIFYYVATTYFRNLPQPLSFVAWPIYWALQGSVLTGVWVIAHECGHHAFSDYQWIDD 120

Gh_A01G2094 ILASLFYYVATNYFHNLPQPLSYVAWPLYWAMQGWILTGVWVIAHECGHHAFSDYQWLDD 114

Gh_D01G1227 ILASLFYHVATNYFPNLPQALSNVAWPLYWAMQGCILTGVWVIAHECGHHAFSDYQWLDD 114

::*..*.:******:*******:*:

Gh_D13G2237 TVGLILHSTLLVPYFSWKISHRRYHSNTGSMERDKVFVPKPKSKLSCFAKYLNNPPGRVL 124

Gh_A13G1850 TVGLILHSALLVPYFSWKISHRRHHSNTGSMERDEVFVPKPKSKLSCFAKYLNNPPGRVL 179

Gh_D13G2238 TVGLILHSALLVPYFSWKISHRRHHSNTGSMERDEVFVPKPKSKLSCFAKYFNNPPGRVL 179

Gh_A01G2091 TIGFILHTFLLTPYFSLKYSHRRHHSNTGSLERDEVFVPKKKSALKWWAKHFNNPLGRFL 180

Gh_D01G1226 TIGFILHTFLLTPYFSLKYSHRRHHSNTGSLERDEVFVPKKKSALKWWAKHFNNPLGRFL 180

Gh_A11G2814 TVGLILHSSLLVPYFSWKYSHRRHHSNTGSLERDEVFVPKKRSSIRWWAKYLNNPPGRFV 180

Gh_D11G3169 TVGLILHSSLLVPYFSWKYSHRRHHSNTGSLERDEVFVPKKRSSIRWWAKYLNNPPGRFV 180

Gh_A01G2094 TVGLILHSSLLVPYFSWKYSHRRHHSNTGSLERDEVFVPKKKSGLRWWAKHFNNPPGRFL 174

Gh_D01G1227 TVGLILHSSLLVPYFSWKYSHRRHHSNTGSLERDEVFVPKKKSGLRWWAKHFNNPPGRFL 174

*:*:***: **.**** * ****:******:***:***** :* : :**::*** **.:

Gh_D13G2237 SLVVTLTLGWPMYLAFNVSGRYYDQLASHYN--GPIFSDCERLQVYISDASIFTVIYVLY 182

Gh_A13G1850 SLVVTLTLGWPMYLAFNVSGRYYDRLASHYNPYGPIYSDRERLQVYISDTGIFAVIYVLY 239

Gh_D13G2238 SLVVTLTLGWPMYLAFNVSGRYYDRLASHYNPYGPIYSERERLQVYISDAGIVAVIYVLY 239

Gh_A01G2091 EISIQLTLGWPLYLLFNITGPPYDS----------------------------------- 205

Gh_D01G1226 EISIQLTLGWPLYLLFNITGPPYDRLACHYDPYGPIFSDRQRLQIYVSDAGVLAVTYALY 240

Gh_A11G2814 TVTIQLTLGWPLYLAFNVAGRPYEGLACHYNPYGPIYNDRERLQIYISDVGVLAVTYGLY 240

Gh_D11G3169 TITIQLTLGWPLYLAFNVAGRPYEGFACHYNPYGPIYNDRERLQIYISDVGVLAVTYGLY 240

Gh_A01G2094 SITIQLTLGWPLYLAFNVAGRPYDRFACHYDPYGPIFSDRERLQIYISDAGVLAVAYALY 234

Gh_D01G1227 SITIQLTLGWPLYLAFNVAGRPYDRFACHYDPYGPIFSDRERLQIYISDAGVLAVAYALY 234

: : ******:** **::* *:

Gh_D13G2237 KITATKGLAWLLCTYGVPLLIVHAFLVLLTLLRVKTQRCFFR--------------YNRE 228

Gh_A13G1850 KIAATKGLAWLLCTYGVPLLIVNAFLVLITYLQHTHSALPHYDSSEWDWLRGALSTMDRD 299

Gh_D13G2238 KIAATKGLAWLLCTYGVPLLIVNAFLVLITYLQHTHSALPHYDSSEWDWFRGALSTIDRD 299

Gh_A01G2091 ------------------------------------------------------------ 205

Gh_D01G1226 LLVLAKGVGWVISVYGVPLLVVNAFLVMITYLQHTHPSLPHYESSEWDWLRGALSTVDRD 300

Gh_A11G2814 RLVLAKGLAWVICVYGVPLLIVNAFLVMITYLQHTHPALPHYDSSEWDWLRGALATVDRD 300

Gh_D11G3169 RLVLAKGLAWVICVYGVPLLIVNAFLVMITYLQHTHPALPHYDSSEWDWLRGALATVDRD 300

Gh_A01G2094 RLVLAKGVGWVISVYGVPLLVVNAFLVMITYLQHTHPSLPHYDSSEWDWMRGALSTVDRD 294

Gh_D01G1227 RLVLAKGVGWVISVYGVPLLVVNAFLVMITYLQHTHPSLPHYDSSEWDWMRGALSTVDRD 294

Gh_D13G2237 YDSLSSVVRT---------------------SNLADRHGPGVSFPFDRTLIYKAMWRKAK 267

Gh_A13G1850 FGVLNKVFHNITDTHVAHHLFSTMPHYHAMEATKAIKPILGKYYPFDGTPIYKAMWREAK 359

Gh_D13G2238 YGVLNKVFHNITDTHVAHHLFSTMPHYHAMEATKAIKPILGKYYPFDGTPIYKAMWREAK 359

Gh_A01G2091 ------------------------------------------------------------ 205

Gh_D01G1226 YGILNKVFHDITRIHVAHHLFPTIPHYHAMEATKVIKPVLGEYYQFDGTPVYKAIWRELK 360

Gh_A11G2814 YGILNKVFHNITDTHVAHHLFSTMPHYHAMEATKAIKPILGEYYSFDGTPVYKAIFREAK 360

Gh_D11G3169 YGILNKVFHNITDTHIAHHLFSTMPHYHAMEATKAIKPILGEYYSFDGTPVYKAIFREAK 360

Gh_A01G2094 YGILNKVFHNITDTHVAHHLFSTMPHYHAMAATKAIKPILGEYYQFDGMPVYKAIWREAK 354

Gh_D01G1227 YGILNKVFHNITDTHVAHHLFSTMPHYHAMVATKAIKPILGEYYQFDGMPVYKAIWREAK 354

Gh_D13G2237 ECLYVEPDVGGG--GSKCVFWYRNKF 291

Gh_A13G1850 ECLYVEPDVGGGGGGSKGVFWYRNKF 385

Gh_D13G2238 ECLYVEADVGG--GGSKGVFWYRNKF 383

Gh_A01G2091 -------------------------- 205

Gh_D01G1226 ECVYVEPDAGD--N-DKGVLWFRNKL 383

Gh_A11G2814 ECIYVEPDEGE--QSSKGVFWFRNKI 384

Gh_D11G3169 ECIYVEPDEGE--QSSKGVFWFRNKI 384

Gh_A01G2094 ECLYVEPDEGD--K-DKGVFWFRNKL 377

Gh_D01G1227 ECLYVEPDEGD--K-DKGVFWFRNKL 377

**(C)**

**Figure S1 The coding sequences and phylogenetic analysis of *GhFAD2***

**A. The coding sequences of *GhFAD2* genes.** The fragment in *GhFAD2-3D* and *GhFAD2-3A* targeted for RNAi is highlighted in red color. The underlined sequence in *GhFAD2-4A* was filled up in this study, which is a gap in the TM-1 genome (Zhang et al. 2015)

**B. Putative members of the *GhFAD2* family in the TM-1 genome identified based on blastp search using the protein sequence of the published GhFAD2-1 (X97016).** Gh_D13G2237 contains three indels (50, 14 and 21 aa, respectively) compared to other proteins, and its 3rd deletion contains the 3rd conserved histidine-cluster observed in all FAD2 protein, this gene was thus considered as a non-functional FAD2 and not analysed further in this study. The three conserved histidine-clusters are highlighted in red. The annotated Gh_A01G2091 was incomplete due to gap in the genomic sequence, and the missing sequence was filled up by sequence cloning in this study.

**C. Phylogenetic analysis of the cotton FAD2 family.** The tree was generated based on protein sequences of cotton FAD2 using the Maximum likelihood module of the MEGA6 software.

**(A)**


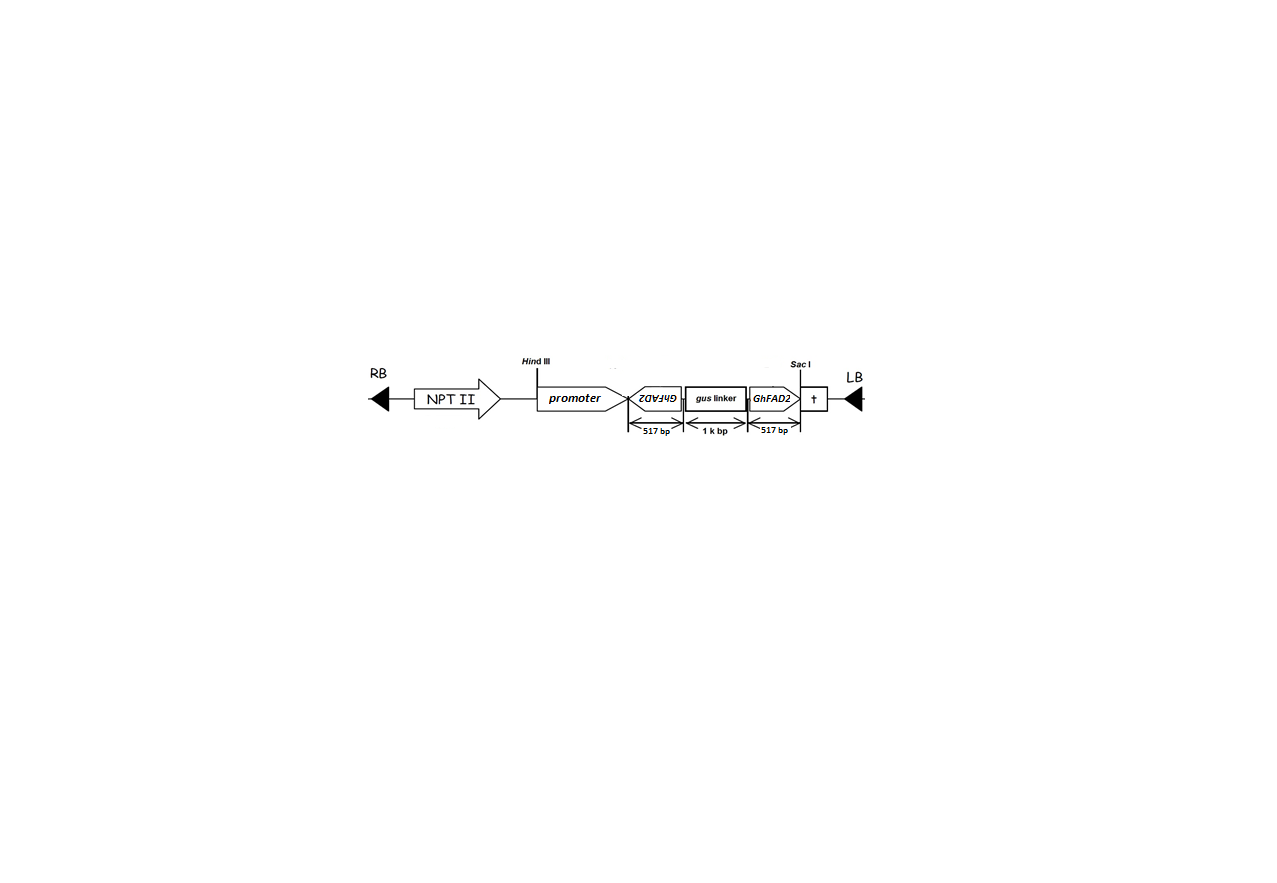


**(B)**


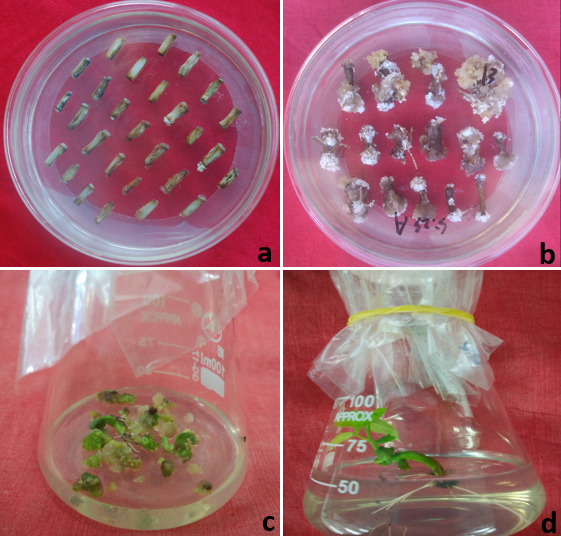


M 1 2 3 4 5 6 7 8 9 10

M 1 2 3 4 5 6 7 8 9 10

**(C)**


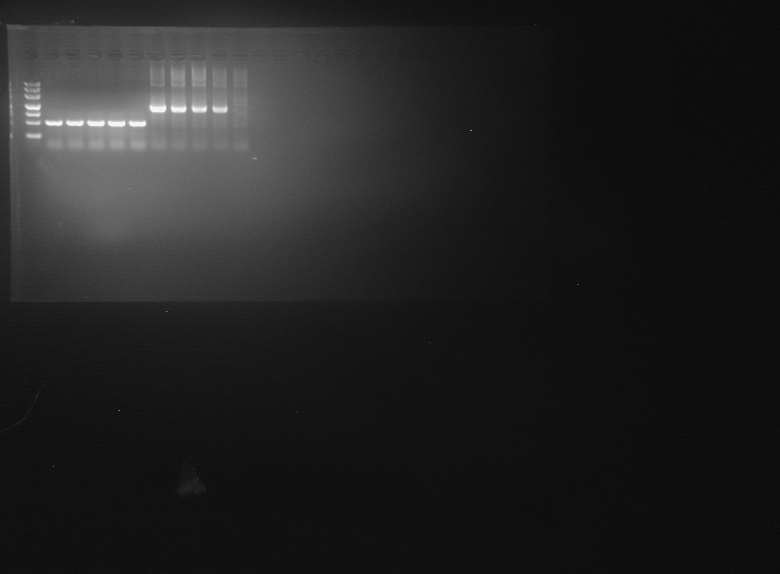


(bp)

4,500

3,000

2,000

1,200

800

500

200

*gus linker-GhFAD2*

*18s rDNA*

**Figure S2 Generation and molecular analysis of transgenic plants**

**A. Schematic representation of the pBIAP-ds*GhFAD2* expression cassettes used for cotton transformation**. promoter, Anther-specific promoter; NPT II, *neomycin phosphotransferase II* gene; *GhFAD2*, The partial fragment *GhFAD2-3D* coding sequence; RB, right border; LB, left border;

**B. Generation of transgenic plants.** a, induction of embryonic calli; b, calli produced from explants; c, embryoid produced from calli; d, regeneration of kanamycin resistant plantlets.

**C. Detection of the *GhFAD2-gus linker* fusion fragment in non-transformed control and transgenic plants by PCR**. A 1,113-bp fusion fragment was amplified and 18s rDNA was served as a control. The primers used in amplification were 5’-CTGTACAGCGAAGAGGCAGTC-3’ and 5’-CGTTGTAGATAGGACCGTAT-3’ for *GhFAD2-gus linker* fusion fragment, and 5’-GAGTCTGGTAATTGGAATGAG-3’ and 5’-TTCGCAGTTGTTCGTCTT-3’ for 18S rDNA. Lane M, DNA marker III; Lane 1-4; 18s rDNA sequence was amplified from transformants line; Lane 5, 18s rDNA sequence was amplified from untransformed control; Lane 6-9, The fusion fragment was amplified from transformants line; Lane 10, The fusion fragment was amplified from untransformed control.

**Table S1 Representative differentially expressed genes involved in anther lipid metabolism**

| Gene category | Gene ID | Genes description | Meiosis stage  (*fad2-3* *vs* WT) | | Tetrad stage  (*fad2-3* *vs* WT) | |
| --- | --- | --- | --- | --- | --- | --- |
|  |  |  | log2(FC) | *P value* | log2(FC) | *P value* |
| Biosynthesis of unsaturated fatty acids | *Gh_A11G2814* | Fatty acid desaturase 2 | -0.63 | 1.95E-10 | -1.89 | 6.02E-17 |
|  | *Gh_D11G3169* |  | -0.31 | 0.0019 | -0.85 | 3.78E-10 |
|  | *Gh_A13G1850* |  | -0.62 | 0.0060 | -2.01 | 1.88E-21 |
|  | *Gh_D13G2238* |  | -2.52 | 6.23E-05 | -5.06 | 3.64E-27 |
|  | *Gh_A01G2094* |  | -- | -- | -1.43 | 8.78E-05 |
|  | *Gh_D01G1226* |  | -- | -- | -1.59 | 1.29E-08 |
|  | *Gh_A09G0848* | Fatty acid desaturase 3 | -1.03 | 0.0003 | -3.60 | 2.39E-17 |
|  | *Gh_A07G0946* |  | 0.42 | 0.0070 | -2.61 | 1.87E-20 |
|  | *Gh_D07G1026* |  | -- | -- | -1.05 | 0.0027 |
|  | *Gh_D09G0870* |  | -- | -- | -3.65 | 2.62E-13 |
|  | *Gh_D05G0430* | Delta(8)-fatty-acid desaturase | -0.49 | 0.0030 | -- | -- |
|  | *Gh_D11G0983* |  | 0.43 | 0.0010 | -- | -- |
|  | *Gh_A11G0840* |  | -0.64 | 0.0030 | -- | -- |
| Linoleic acid metabolism and alpha-Linolenic  acid metabolism | *Gh_D06G2175* | Lipoxygenase 3 | -- | -- | -4.28 | 0.0003 |
|  | *Gh_D02G1542* | Lipoxygenase 6 | -0.48 | 0.0030 | -- | -- |
|  | *Gh_D08G2225* | Linoleate 9S-lipoxygenase | 3.91 | 0.0013 | -- | -- |
|  | *Gh_A09G2247* | Linoleate 9S-lipoxygenase 5 | 0.56 | 0.0062 | 3.07 | 4.31E-19 |
|  | *Gh_D09G2080* |  | 0.51 | 0.0007 | 1.31 | 1.25E-22 |
|  | *Gh_D07G1987* | Probable linoleate 9S-lipoxygenase 4 | 4.36 | 1.18E-23 | -- | -- |
|  | *Gh_A08G1864* | Probable linoleate 9S-lipoxygenase 5 | 4.60 | 0.0042 | -- | -- |
|  | *Gh_A13G0888* |  | -- | -- | 0.97 | 2.70E-07 |
|  | *Gh_D05G0683* | Linoleate 13S-lipoxygenase 2-1 | -0.54 | 0.0021 | 1.00 | 6.11E-08 |
|  | *Gh_A10G0504* | Linoleate 13S-lipoxygenase 3-1 | 1.34 | 0.0005 | 1.10 | 2.41E-12 |
|  | *Gh_D10G2595* |  | 1.52 | 0.0002 | 1.24 | 9.65E-12 |
| Cutin, suberine and wax biosynthesis | *Gh_D04G1447* | Cytochrome P450 86B1 | 1.35 | 1.05E-07 | -- | -- |
|  | *Gh_A04G0930* |  | 1.16 | 1.34E-07 | -- | -- |
|  | *Gh_A13G0518* |  | -- | -- | -1.30 | 1.89E-12 |
|  | *Gh_A03G2129* |  | 1.43 | 8.66E-05 | 4.06 | 3.04E-08 |
|  | *Gh_D02G1587* |  | 0.56 | 5.66E-05 | 1.41 | 0.0001 |
|  | *Gh_A12G1506* | Cytochrome P450 704B1 | 4.33 | 1.20E-99 | -- | -- |
|  | *Gh_D12G2768* |  | 4.70 | 1.10E-70 | -- | -- |
|  | *Gh_D08G1463* | Cytochrome P450 86A8 | -- | -- | 1.37 | 0.0030 |
|  | *Gh_A08G1178* |  | -- | -- | 3.11 | 1.69E-10 |
|  | *Gh_A11G0806* |  | -0.54 | 5.77E-05 | -- | -- |
|  | *Gh_A12G2100* |  | -1.46 | 0.0062 | -1.39 | 2.30E-07 |
|  | *Gh_D08G1972* |  | -- | -- | -1.85 | 0.0008 |
|  | *Gh_D12G2271* |  | -- | -- | -0.77 | 9.41E-06 |
|  | *Gh_D10G0552* | ω-hydroxyacid dehydrogenase | -0.34 | 0.0014 | 0.92 | 4.54E-10 |
|  | *Gh_D05G1294* |  | -0.89 | 1.66E-09 | 1.07 | 2.18E-06 |
|  | *Gh_A05G1124* |  | -1.08 | 2.38E-10 | -- | -- |
|  | *Gh_A10G2254* |  | -- | -- | 0.85 | 5.83E-08 |
|  | *Gh_A08G1604* |  | -0.62 | 2.47E-06 | -- | -- |
|  | *Gh_A11G0078* |  | 3.06 | 5.78E-15 | -2.25 | 2.11E-29 |
|  | *Gh_D08G1915* |  | -0.73 | 3.12E-08 | -- | -- |
|  | *Gh_D11G0083* |  | 1.52 | 3.35E-06 | -1.25 | 1.15E-22 |
|  | *Gh_D12G2232* |  | 7.69 | 4.80E-67 | -- | -- |
|  | *Gh_A12G2054* |  | 6.15 | 1.26E-57 | -- | -- |
|  | *Gh_A06G1063* | Fatty acid 2-hydroxylase 1 | -1.92 | 0.0080 | -3.45 | 4.19E-27 |
|  | *Gh_A06G1603* |  | -0.44 | 7.74E-05 | -- | -- |
|  | *Gh_A02G0903* |  | -- | -- | 1.24 | 2.12E-05 |
|  | *Gh_D06G1958* |  | -- | -- | 0.91 | 1.58E-13 |
|  | *Gh_A09G0895* | Alcohol-forming fatty acyl-CoA reductase | -1.66 | 7.24E-05 | -2.84 | 1.85E-09 |
|  | *Gh_D09G0571* | Peroxygenase 1 | -2.40 | 5.34E-08 | -4.82 | 4.39E-46 |
|  | *Gh_A09G0571* |  | -2.09 | 1.11E-05 | -3.35 | 3.76E-29 |
|  | *Gh_A10G0978* | Probable peroxygenase 3 | 0.71 | 0.0025 | -- | -- |
|  | *Gh_A10G0977* |  | 0.61 | 3.56E-09 | -- | -- |
|  | *Gh_D09G0572* |  | -1.46 | 0.0002 | -- | -- |
|  | *Gh_D10G1585* |  | -- | -- | -0.88 | 3.66E-09 |
|  | *Gh_D10G1584* |  | -- | -- | -0.77 | 3.59E-07 |
|  | *Gh_A09G0574* |  | -- | -- | 0.67 | 0.0020 |
|  | *Gh_A02G0527* | Aldehyde dehydrogenase family | -0.48 | 0.0020 | -0.53 | 7.16E-05 |
|  | *Gh_A02G1616* |  | 1.83 | 1.07E-17 | -- | -- |
|  | *Gh_A05G0568* |  | 0.82 | 6.94E-05 | 1.27 | 1.61E-24 |
|  | *Gh_A06G0526* |  | 1.68 | 0.0016 | -- | -- |
|  | *Gh_A06G1256* |  | 0.35 | 0.004497 | -- | -- |
|  | *Gh_D03G0106* |  | 0.97 | 6.57E-10 | -0.66 | 3.90E-09 |
|  | *Gh_D05G0697* |  | 0.81 | 2.44E-05 | -- | -- |
|  | *Gh_D05G2245* |  | -1.48 | 2.92E-08 | -1.91 | 7.57E-06 |
|  | *Gh_D06G0414* |  | -0.79 | 1.46E-05 | -0.96 | 0.000571 |
|  | *Gh_D06G0580* |  | 0.85 | 0.0008 | -- | -- |
|  | *Gh_D06G1578* |  | 0.42 | 0.0003 | -- | -- |
|  | *Gh_A05G0157* |  | -- | -- | 1.43 | 1.13E-45 |
|  | *Gh_D02G0793* |  | -- | -- | -0.36 | 0.0010 |
|  | *Gh_D05G0221* |  | -- | -- | 0.82 | 5.27E-10 |
|  | *Gh_A06G1389* | Protein ECERIFERUM 1 | -- | -- | 0.96 | 8.16E-09 |
|  | *Gh_D04G0694* |  | -1.80 | 5.13E-28 | -- | -- |
|  | *Gh_A09G1215* | Fatty acyl-CoA reductase 2 | -0.72 | 0.0006 | -1.17 | 5.89E-08 |
|  | *Gh_D09G1221* |  | -1.36 | 5.47E-08 | -- | -- |
|  | *Gh_A01G0664* | Long-chain-alcohol O-fatty-acyltransferase | -3.74 | 0.0014 | -- | -- |
|  | *Gh_A13G1146* | Probable long-chain-alcohol O-fatty-acyltransferase 1 | -0.50 | 0.0016 | -- | -- |
|  | *Gh_D06G1688* | Probable long-chain-alcohol O-fatty-acyltransferase 4 | 0.57 | 6.42E-05 | -- | -- |
|  | *Gh_D13G1428* | Probable long-chain-alcohol O-fatty-acyltransferase 5 | -3.58 | 1.98E-10 | -- | -- |

Gene IDs and descriptions were based on Zhang et al. (2015). “--” represents no significant difference. FC, fold change.

**Table S2 Primers of selected genes used for qRT-PCR analysis**

| Gene ID | Predict coding protein | Primer sequence (5’-3’) | Product length (bp) |
| --- | --- | --- | --- |
| Gh_D10G1199 | Acyl-[acyl-carrier-protein] desaturase | F: CGGCAATGGCTACCTTC  R: CTTATCTGGACGAGGCATAT | 110 |
| Gh_D11G3169 | Fatty acid desaturase 2 | F: CGTCACAATCACCATTCAG  R: CGTTGTAGATAGGACCGTAT | 119 |
| Gh_A09G0848 | Omega-3 fatty acid desaturase | F: CTGTAATCGGTCCATCCAT  R: GCTTCTGCTCGTATCCAT | 111 |
| Gh_D04G1447 | Cytochrome P450 86B1 | F: GGAACCTTGAGCATCTTCT  R: GTCGTCGGCGTTGAATA | 110 |
| Gh_D12G2768 | Cytochrome P450 704B1 | F: AGATGGAACCAGAGGAACA  R: GATGCGGACAGGTATTCG | 119 |
| Gh_A10G2254 | ω-hydroxyacid dehydrogenase | F: CAGTGTATTGATGTTGGAGAG  R: CAAGGCGATGTGGAAGTT | 82 |
| Gh_D06G1578 | ω-oxo-acid dehydrogenase | F: GCACACTTCTTCTTCAAGG  R: CTATTATACCGCCACCAGTA | 92 |
| Gh_A10G0978 | Peroxygenase | F: GGAGTTGTGGCAGATGAC  R: CTTGTCCTTGGCTACATAATAC | 106 |
| Gh_D09G1325 | Cytochrome P450 94C1 | F: TGACGACGACATATTACCA  R: TTGAACTCCAAGCAATCTG | 117 |

Gene IDs and coding protein were based on Zhang et al. (2015).
